# Trinuclear nickel coordination complexes of phenanthrene-9,10-dione dioxime

**DOI:** 10.1107/S2056989016004023

**Published:** 2016-03-24

**Authors:** Owen M. Williams, Alan H. Cowley

**Affiliations:** a 105 E. 24th St., Austin, TX 78704, USA

**Keywords:** crystal structure, nickel, H_2_pqd, α–β dioxime, trinuclear, hydrogen bonding

## Abstract

A trinuclear nickel complex of phenanthrene-9,10-dione dioxime (H_2_pqd), namely bis­[μ_2_-9,10-bis­(oxido­imino)­phenanthrene]­bis­[μ_2_-10-(oxido­imino)phenanthrene-9-one oxime](phenanthrene-9,10-dione dioxime)trinickel(II) toluene disolvate, [Ni_3_(C_14_H_8_N_2_O_2_)_2_(C_14_H_9_N_2_O_2_)_2_(C_14_H_10_N_2_O_2_)]·2C_7_H_8_, has been isolated and its crystal structure determined. This complex features three independent Ni^II^ atoms that are arranged in a triangular fashion along with five supporting ligands. There are two square-planar Ni^II^ atoms and a third pseudo-octa­hedral Ni^II^ atom. While the square-planar Ni^II^ atoms are stacked, there are no ligand bridges between them. Each square-planar Ni^II^ atom, however, bridges with the pseudo-octa­­hedral Ni^II^ atom through Ni—N—O—Ni and Ni—O—Ni bonds. A fluorido­bor­ation reaction of the proton-bridged species gave the analogous complex bis­(μ_2_-bis­{[10-(oxido­imino)-9,10-di­hydro­phenanthren-9-yl­idene]amino}di­fluorido­borato)(phenanthrene-9,10-dione dioxime)trinickel(II) dichloromethane trisolvate, [Ni_3_(C_28_H_16_BF_2_N_4_O_2_)_4_(C_14_H_10_N_2_O_2_)]·3CH_2_Cl_2_, which shows the same binding structure, but features a widened Ni—Ni inter­action between the square-planar Ni^II^ atoms. The proton-bridged complex completes the macrocyclic coordination around the square-planar Ni^II^ atoms by means of an O—H⋯O hydrogen bond. Both compounds feature O—H⋯N hydrogen bonds between the oxime and the N atoms attached to square-planar nickel atom. The nickel units show no direct inter­action with their nearest neighbors in the extended lattice. Two π-stacking inter­actions between adjacent mol­ecules are found: one with a centroid–centroid distance of 3.886 (2) Å and the other with a centroid–centroid distance of 4.256 (3) Å. In the latter case, although not aromatic, the distance to the centroid of the central phenanthrene ring is shorter, with a distance of 3.528 (3) Å. Toluene mol­ecules occupy the solvent channels that are oriented along the *c* axis. In the fluorido­boronate structure, the solvent (DCM) was too badly disordered to be modelled, so its contribution was removed using SQUEEZE [Spek (2015 ▸). *Acta Cryst.* C**71**, 9–18].

## Abstract 2

Trinuclear nickel complexes have been isolated and characterized. These complexes resulted from reaction with the constrained α–β dioxime congener of phenanthrene-9,10-dione.

## Chemical context   

Oxime functional groups can coordinate to transition metal ions in a variety of ways, due to the presence of both nitro­gen and oxygen donors. On account of the multitude of possible coordinations, these ligands, and particularly α–β dioximes, have the capability of forming bridging multinuclear complexes with many transition metals, including nickel (Chaudhuri, 2003 ▸). From the standpoint of single-mol­ecule magnets, these multi-nuclear complexes play an important role due to their ability to facilitate spin-frustration in magnetic transition-metal clusters (Aromí & Brechin, 2006 ▸). Other nickel polynuclear compounds supported by oxime ligands have been reported (Jiang *et al.*, 2005 ▸; Biswas *et al.*, 2009 ▸).

Phenanthrene­quinone dioxime (pqdH_2_) is an α–β dioxime ligand that incorporates a constrained ring system. Similar to other dioximes, however, it exists as three separate stereoisomers (*E*–*E*, *E*–*Z*, and *Z*–*Z*), as confirmed by liquid chromatography – mass spectrometry. Inter­estingly, although this compound was synthesized over 100 years ago (Schmidt & Söll, 1907 ▸), no coordination complexes of this ligand have been structurally characterized to date.
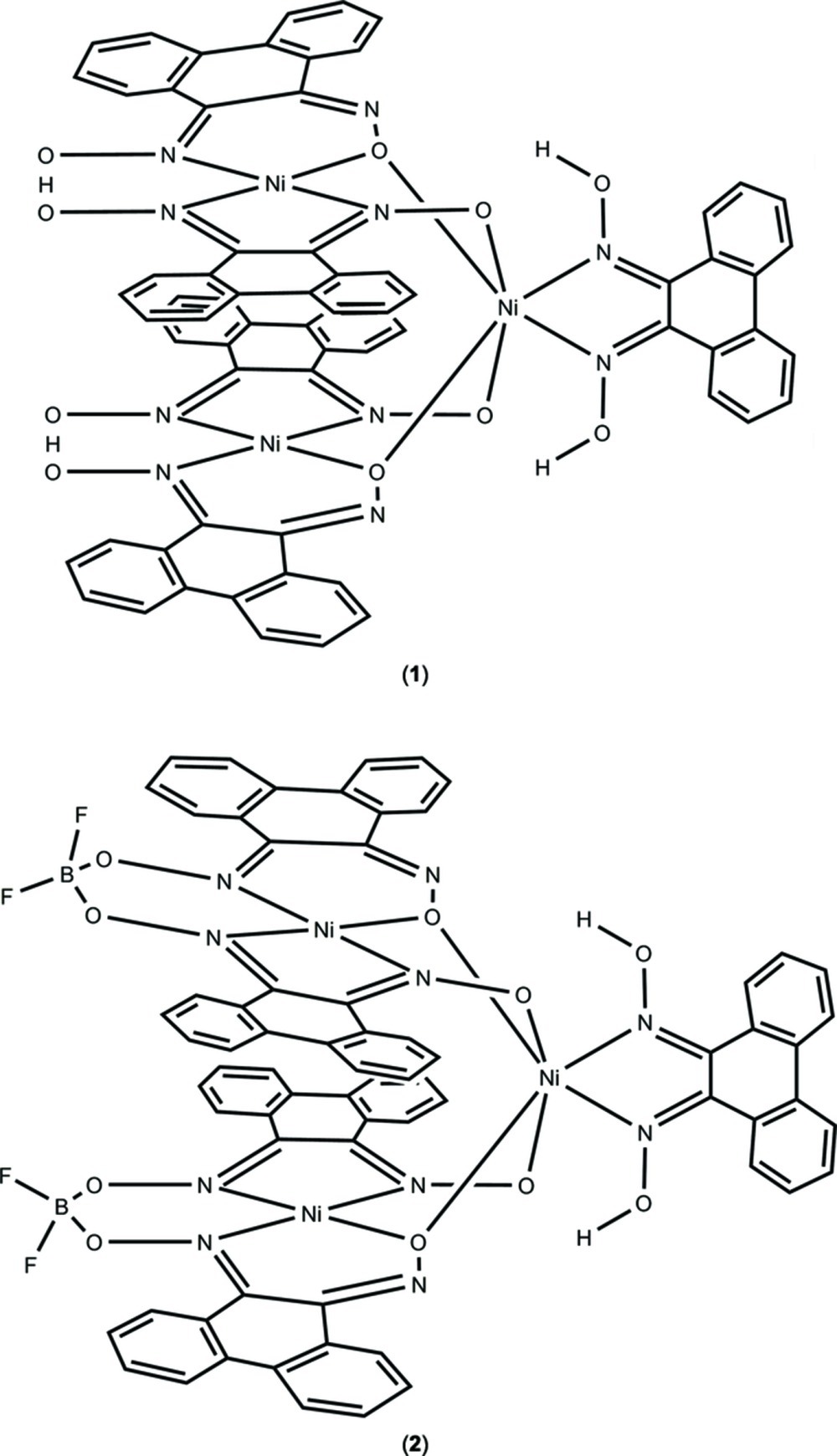


## Structural commentary   

**Figure 1:**
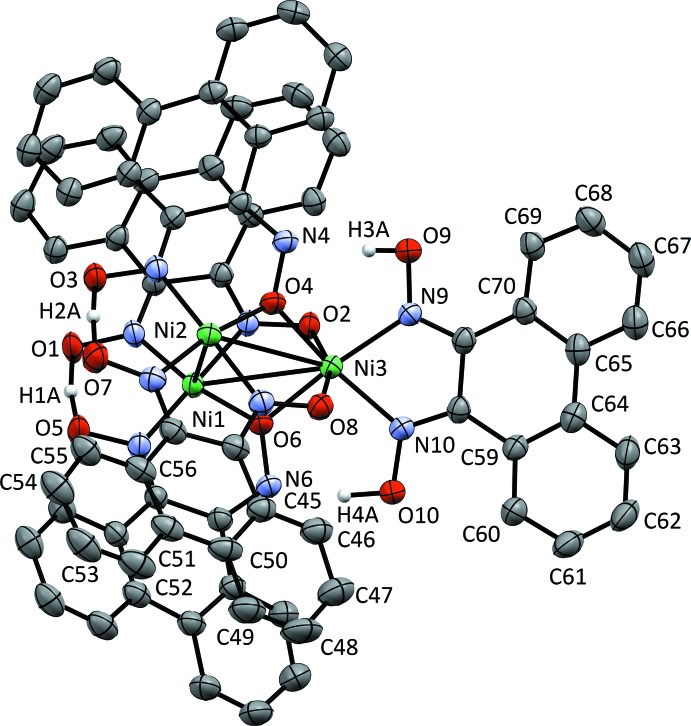
Displacement ellipsoid plot at the 50% probability level for [Ni_3_(H_2_pqd)(Hpqd)_2_(pqd)_2_]. H atoms (with the exception of hydrogen-bonded atoms) and solvent mol­ecules have been omitted for clarity.

Fig. 1 ▸ shows the structure of [Ni_3_(H_2_pqd)(Hpqd)_2_(pqd)_2_], (**1**). This complex consists of three Ni^II^ atoms in a triangular arrangement, two of which are in a square-planar coordination environment, while the third is in a pseudo-octa­hedral coord­ination environment. The square-planar Ni^II^ atoms (Ni1 and Ni2) consist of one *N*,*N*-coordinating and one *N*,*O*-coordinating ligand. These ligands form bridges with the pseudo-octa­hedral Ni^II^ atom (Ni3) by means of their oxime O atoms. This arrangement permits the formation of Ni—N—O—Ni and Ni—O—Ni bridges between each square-planar Ni^II^ atom and the pseudo-octa­hedral Ni^II^ atom.

The structural features of the core ligation sphere warrant special attention. The Ni_sp_—Ni_sp_ distance is 3.3657 (9) Å, a distance that precludes the presence of any metal–metal bonding. However, the distances between each of these nickel moieties and the pseudo-octa­hedral Ni^II^ atom are nearly identical [Ni_sp_—Ni_oct_ = 3.2697 (7), 3.2674 (7) Å]. The pseudo-octa­hedral nickel geometry deviates significantly from a perfect octa­hedral symmetry [O2—Ni3—O8 = 160.00 (9), O4—Ni3—N10 = 164.8 (1), N9—Ni3—O6 = 165.5 (1)°].

**Figure 2:**
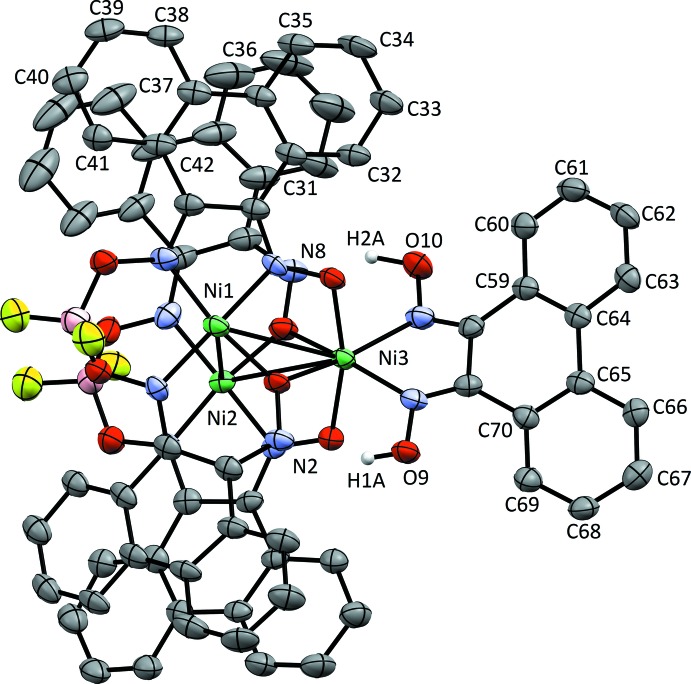
Displacement ellipsoid plot at the 50% probability level of [Ni_3_(H_2_pqd)(BF_2_pqd)_2_(pqd)_2_]. H atoms (with the exception of hydrogen-bonded atoms) and solvent mol­ecules have been omitted for clarity.

Fig. 2 ▸ shows the complete coordination geometry of the compound [Ni_3_(pqdH_2_)(pqdBF_2_)_2_(pqd)_2_], (**2**). The physical arrangement of the ligation sphere directly mimics that of (**1**). In this case, however, the steric bulk of the BF_2_ groups forces an expansion of the stacked square-planar nickel units, resulting in an Ni_sp_—Ni_sp_ distance of 3.592 (1) Å. The distances between these units and the pseudo-octa­hedral Ni^II^ atom, however, remain similar [Ni_sp_—Ni_oct_ = 3.274 (1), 3.255 (1) Å]. Overall, the entire structure retains all the other structural features that are present in the proton-bridged compound.

The phenanthrene backbones show pronounced twisting between their aromatic rings, which precludes conjugation across this unit. For the proton-bridged complex, the angle between mean planes within a single phenanthrene backbone ranges from 9.24 (19)° (between C45–C50 and C51–C56) to 15.44 (13)° (between C59–C64 and C65–C70). For the BF_2_-bridged complex, there is a wider range of angles, with 5.2 (4)° (between C31–C36 and C37–C42) being the smallest, and 17.5 (3)° (between C59–C64 and C65–C70) being the largest.

## Supra­molecular features   

**Figure 3:**
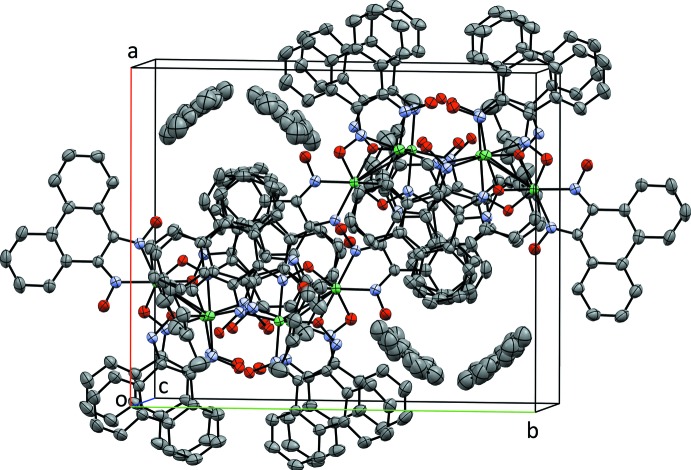
Packing diagram of [Ni_3_(H_2_pqd)(Hpqd)_2_(pqd)_2_], viewed approximately down the *c*-axis direction.

The proton-bridged complex completes the macrocyclic coordination around the square-planar Ni^II^ atoms by means of hydrogen bonds. Furthermore, the ligand that coordinates the pseudo-octa­hedral Ni^II^ atom features hydrogen-bonding inter­actions (Table 1 ▸) between the oxime hydroxy groups and the ligands of the square-planar Ni^II^ atoms. The nickel units show no direct inter­action with their nearest neighbors in the extended lattice. Some π-stacking between adjacent mol­ecules is, however, evident (Fig. 3 ▸). Two inter­actions were found, one with a centroid–centroid distance of 3.886 (2) Å (symmetry code: 1 − *x*, −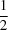 + *y*, 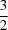 − *z*) and the other with a centroid–centroid distance of 4.256 (3) Å (symmetry code: −*x*, −*y*, 2 − *z*). In the latter case, although not aromatic, the distance to the centroid of the central ring of phenanthrene is shorter, with a distance of 3.528 (3) Å. Toluene mol­ecules occupy the solvent channels that are oriented along the *c* axis.

**Figure 4:**
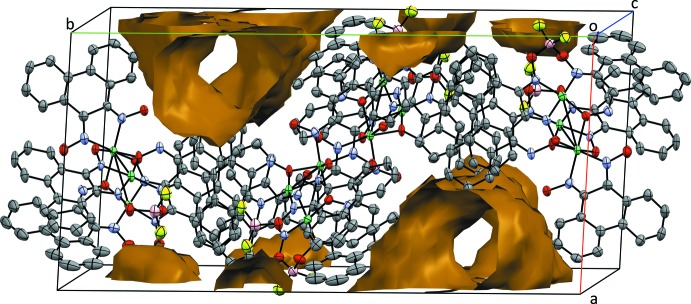
Packing diagram of Ni_3_(H_2_pqd)(BF_2_pqd)_2_(pqd)_2_, viewed approximately down the *c*-axis direction. Voids presented in brown were calculated in *Mercury* (Macrae *et al.*, 2006 ▸) using a probe radius of 1.2 Å.

The BF_2_-bridged complex completes the macrocyclic coordin­ation around the square-planar Ni^II^ atoms by means of covalent O—B—O bonds. However, the hydrogen-bonding inter­actions (Table 2 ▸) that lock the pseudo-octa­hedral Ni^II^ atom remain in place. The nickel units show no direct inter­action with their nearest neighbors in the extended lattice. A solvent channel oriented along the *c* axis is also evident (Fig. 4 ▸). However, the extreme disorder of the solvent does not permit the determination of a suitable model.

## Synthesis and crystallization   

The parent ligand, pqdH_2_ (0.75 g; 3.1 mmol), was dissolved in 100 ml of ethanol, to which nickel(II) acetate (0.33 g, 1.3 mmol) was added. A red precipitate began to form after approximately 30 min. The solution was then allowed to stir for 1 h, followed by cooling in a freezer and filtration of the crude product (yield: 272 mg, 0.2 mmol, 32%). The resulting product was dissolved in DMF solution and layered with toluene, resulting in the formation of crystals of [Ni_3_(H_2_pqd)(Hpqd)_2_(pqd)_2_] after a period of 3–4 d. The crystals grew as red blocks with an asymmetric unit consisting of a complete [Ni_3_(H_2_pqd)(Hpqd)_2_(pqd)_2_] mol­ecule and two toluene solvent mol­ecules.

The foregoing complex is stable enough to undergo a fluorido­boration reaction with boron trifluoride, thereby affording the compound [Ni_3_(H_2_pqd)(BF_2_pqd)_2_(pqd)_2_]. [Ni_3_(H_2_pqd)(Hpqd)_2_(pqd)_2_] was diluted in diethyl ether, thereby creating a slurry. One ml of 1.0 molar BF_3_–OEt_2_ (in ether) was then added and the mixture was allowed to react overnight. The resulting precipitate was then filtered off and washed thoroughly with EtOH and Et_2_O. The resulting precipitate was then dissolved in dichloromethane (DCM) and filtered through Celite (yield: 43 mg, 30 µmol, 79%). Subsequently, a crop of red block-shaped crystals was grown by solvent evaporation over a period of one day.

## Refinement   

Crystal data, data collection and structure refinement details are summarized in Table 3 ▸. In proton-bridged structure (1), atoms H1*A*, H2*A* and H4*A* were found by assignment of difference map peaks and refined isotropically without geometrical constraints. The proton H3*A* was initially placed with the *SHELXL* HFIX 147 command (refinement on rotation) on O9, but was refined freely. Four distinct hydrogen-bonding inter­actions were evident in the trinuclear cluster. Finally, there were two O—H—O inter­actions between an oxime and oximato of each [Ni(Hpqd)(pqd)]^−^ unit that could not be resolved due to rapid conversion to [Ni(pqd)(Hpqd)]^−^. All the restraints that are reported were included for the modelling of the disordered toluene solvent mol­ecules.

In the case of BF_2_-bridged structure (2), atoms H1*A* and H2*A* were affixed to O9 and O10, respectively. They were then refined isotropically without rotational constraints. The SQUEEZE routine (Spek, 2015 ▸) as implemented in *PLATON* (Spek, 2009 ▸) was used to remove the electron density of three solvent DCM mol­ecules per unit cell (calculated: 134 e^−^; 593 Å^3^).

## Supplementary Material

Crystal structure: contains datablock(s) global, 1, 2. DOI: 10.1107/S2056989016004023/pk2575sup1.cif

Structure factors: contains datablock(s) 1. DOI: 10.1107/S2056989016004023/pk25751sup3.hkl

Structure factors: contains datablock(s) 2. DOI: 10.1107/S2056989016004023/pk25752sup2.hkl

CCDC references: 1462814, 1462813

Additional supporting information:  crystallographic information; 3D view; checkCIF report

## Figures and Tables

**Table 1:** Hydrogen-bond geometry (Å, °) for (**1**)

*D*—H⋯*A*	*D*—H	H⋯*A*	*D*⋯*A*	*D*—H⋯*A*
O1—H1*A*⋯O5	1.29 (6)	1.13 (6)	2.405 (4)	169 (6)
O3—H2*A*⋯O7	1.17 (5)	1.23 (5)	2.402 (4)	176 (5)
O9—H3*A*⋯N4	0.86 (6)	1.82 (6)	2.672 (4)	175 (7)
O10—H4*A*⋯N6	1.04 (5)	1.63 (5)	2.657 (4)	168 (4)

**Table 2:** Hydrogen-bond geometry (Å, °) for (**2**)

*D*—H⋯*A*	*D*—H	H⋯*A*	*D*⋯*A*	*D*—H⋯*A*
O9—H1*A*⋯N2	0.85	1.96	2.771 (6)	158
O10—H2*A*⋯N8	1.06	1.77	2.765 (6)	155

**Table 3:** Experimental details

	(**1**)	(**2**)
Crystal data		
Chemical formula	[Ni_3_(C_14_H_8_N_2_O_2_)_2_(C_14_H_9_N_2_O_2_)_2_(C_14_H_10_N_2_O_2_)]·2C_7_H_8_	[Ni_3_(C_28_H_16_BF_2_N_4_O_2_)_4_(C_14_H_10_N_2_O_2_)]·3CH_2_Cl_2_
*M*_r_	1545.55	1456.88
Crystal system, space group	Monoclinic, *P*2_1_/*c*	Monoclinic, *P*2_1_/*c*
Temperature (K)	100	100
*a*, *b*, *c* (Å)	15.973 (3), 18.639 (3), 22.785 (4)	15.6414 (8), 30.8358 (11), 14.7380 (8)
β (°)	101.757 (4)	112.411 (6)
*V* (Å^3^)	6641.1 (19)	6571.5 (6)
*Z*	4	4
Radiation type	Mo *K*α	Cu *K*α
μ (mm^−1^)	0.92	1.67
Crystal size (mm)	0.16 × 0.11 × 0.11	0.29 × 0.07 × 0.04

Data collection		
Diffractometer	Rigaku Saturn724+	Agilent SuperNova Dual Source diffractometer with an Atlas detector
Absorption correction	Multi-scan (*ABSCOR*; Higashi, 1995 ▸)	Multi-scan (*CrysAlis PRO*; Agilent, 2014 ▸)
*T*_min_, *T*_max_	0.799, 1.000	0.303, 1.000
No. of measured, independent and observed [*I* > 2σ(*I*)] reflections	79592, 15260, 13993	25337, 13041, 8782
*R*_int_	0.054	0.060
(sin θ/λ)_max_ (Å^−1^)	0.650	0.626

Refinement		
*R*[*F* ^2^ > 2σ(*F* ^2^)], *wR*(*F* ^2^), *S*	0.067, 0.151, 1.21	0.077, 0.226, 1.03
No. of reflections	15260	13041
No. of parameters	1046	896
No. of restraints	291	0
H-atom treatment	H atoms treated by a mixture of independent and constrained refinement	H atoms treated by a mixture of independent and constrained refinement
Δρ_max_, Δρ_min_ (e Å^−3^)	0.56, −0.68	1.16, −0.83

Computer programs: *CrystalClear* (Rigaku Inc, 2008 ▸), *CrysAlis PRO* (Agilent, 2014 ▸), *SIR2004* (Burla *et al.*, 2005 ▸), *OLEX2* (Dolomanov *et al.*, 2009 ▸), *SHELXL2013* (Sheldrick, 2015 ▸), *Mercury* (Macrae *et al.*, 2006 ▸), *publCIF* (Westrip, 2010 ▸) and *WinGX* (Farrugia, 1999 ▸).

## supplementary crystallographic information

### (1) Bis[µ2-9,10-bis(oxidoimino)phenanthrene]bis[µ2-10-(oxidoimino)phenanthrene-9-one oxime](phenanthrene-9,10-dione dioxime)trinickel(II) toluene disolvate Crystal data

**Table:**

[Ni_3_(C_14_H_8_N_2_O_2_)_2_(C_14_H_9_N_2_O_2_)_2_(C_14_H_10_N_2_O_2_)]·2C_7_H_8_	*F*(000) = 3192
*M**_r_* = 1545.55	*D*_x_ = 1.546 Mg m^−^^3^
Monoclinic, *P*2_1_/*c*	Mo *K*α radiation, λ = 0.71069 Å
*a* = 15.973 (3) Å	Cell parameters from 20424 reflections
*b* = 18.639 (3) Å	θ = 1.3–31.1°
*c* = 22.785 (4) Å	µ = 0.92 mm^−^^1^
β = 101.757 (4)°	*T* = 100 K
*V* = 6641.1 (19) Å^3^	Block, red
*Z* = 4	0.16 × 0.11 × 0.11 mm

### (1) Bis[µ2-9,10-bis(oxidoimino)phenanthrene]bis[µ2-10-(oxidoimino)phenanthrene-9-one oxime](phenanthrene-9,10-dione dioxime)trinickel(II) toluene disolvate Data collection

**Table:**

Rigaku Saturn724+ (2x2 bin mode) diffractometer	15260 independent reflections
Radiation source: Sealed Tube	13993 reflections with *I* > 2σ(*I*)
Detector resolution: 28.5714 pixels mm^-1^	*R*_int_ = 0.054
dtprofit.ref scans	θ_max_ = 27.5°, θ_min_ = 1.8°
Absorption correction: multi-scan (ABSCOR; Higashi, 1995)	*h* = −20→20
*T*_min_ = 0.799, *T*_max_ = 1.000	*k* = −24→24
79592 measured reflections	*l* = −29→29

### (1) Bis[µ2-9,10-bis(oxidoimino)phenanthrene]bis[µ2-10-(oxidoimino)phenanthrene-9-one oxime](phenanthrene-9,10-dione dioxime)trinickel(II) toluene disolvate Refinement

**Table:**

Refinement on *F*^2^	291 restraints
Least-squares matrix: full	Hydrogen site location: mixed
*R*[*F*^2^ > 2σ(*F*^2^)] = 0.067	H atoms treated by a mixture of independent and constrained refinement
*wR*(*F*^2^) = 0.151	*w* = 1/[σ^2^(*F*_o_^2^) + (0.0495*P*)^2^ + 7.4732*P*] where *P* = (*F*_o_^2^ + 2*F*_c_^2^)/3
*S* = 1.21	(Δ/σ)_max_ = 0.001
15260 reflections	Δρ_max_ = 0.56 e Å^−^^3^
1046 parameters	Δρ_min_ = −0.68 e Å^−^^3^

### (1) Bis[µ2-9,10-bis(oxidoimino)phenanthrene]bis[µ2-10-(oxidoimino)phenanthrene-9-one oxime](phenanthrene-9,10-dione dioxime)trinickel(II) toluene disolvate Special details

**Table:**

Geometry. All esds (except the esd in the dihedral angle between two l.s. planes) are estimated using the full covariance matrix. The cell esds are taken into account individually in the estimation of esds in distances, angles and torsion angles; correlations between esds in cell parameters are only used when they are defined by crystal symmetry. An approximate (isotropic) treatment of cell esds is used for estimating esds involving l.s. planes.

### (1) Bis[µ2-9,10-bis(oxidoimino)phenanthrene]bis[µ2-10-(oxidoimino)phenanthrene-9-one oxime](phenanthrene-9,10-dione dioxime)trinickel(II) toluene disolvate Fractional atomic coordinates and isotropic or equivalent isotropic displacement parameters (Å^2^)

**Table:**

	*x*	*y*	*z*	*U*_iso_*/*U*_eq_	Occ. (<1)
C1	0.3573 (2)	0.26380 (17)	0.77353 (15)	0.0300 (7)	
C2	0.4143 (2)	0.20289 (17)	0.79218 (14)	0.0287 (6)	
C3	0.5058 (2)	0.21463 (17)	0.81436 (14)	0.0289 (7)	
C4	0.5622 (2)	0.15956 (19)	0.83987 (15)	0.0341 (7)	
H4	0.5433	0.1123	0.8376	0.041*	
C5	0.6447 (2)	0.1749 (2)	0.86803 (16)	0.0392 (8)	
H5	0.6811	0.1381	0.8850	0.047*	
C6	0.6738 (2)	0.2450 (2)	0.87123 (17)	0.0423 (9)	
H6	0.7292	0.2554	0.8913	0.051*	
C7	0.6209 (2)	0.2991 (2)	0.84477 (17)	0.0412 (8)	
H7	0.6418	0.3458	0.8465	0.049*	
C8	0.5364 (2)	0.28614 (18)	0.81521 (15)	0.0318 (7)	
C9	0.4809 (2)	0.34500 (17)	0.78633 (15)	0.0306 (7)	
C10	0.5151 (2)	0.41244 (19)	0.77676 (16)	0.0371 (8)	
H10	0.5735	0.4200	0.7897	0.045*	
C11	0.4652 (2)	0.46755 (19)	0.74890 (17)	0.0403 (8)	
H11	0.4897	0.5119	0.7442	0.048*	
C12	0.3783 (2)	0.45710 (18)	0.72779 (17)	0.0388 (8)	
H12	0.3445	0.4941	0.7085	0.047*	
C13	0.3423 (2)	0.39116 (17)	0.73569 (16)	0.0334 (7)	
H13	0.2841	0.3841	0.7212	0.040*	
C14	0.3922 (2)	0.33465 (16)	0.76530 (15)	0.0294 (7)	
C15	0.3654 (2)	0.24577 (17)	0.93609 (14)	0.0292 (7)	
C16	0.4342 (2)	0.19229 (17)	0.94884 (14)	0.0283 (6)	
C17	0.5203 (2)	0.21590 (17)	0.98016 (14)	0.0293 (7)	
C18	0.5819 (2)	0.16511 (18)	1.00548 (14)	0.0327 (7)	
H18	0.5684	0.1165	1.0024	0.039*	
C19	0.6622 (2)	0.1864 (2)	1.03489 (15)	0.0364 (8)	
H19	0.7032	0.1524	1.0507	0.044*	
C20	0.6815 (2)	0.2591 (2)	1.04065 (17)	0.0410 (8)	
H20	0.7354	0.2738	1.0606	0.049*	
C21	0.6211 (2)	0.3092 (2)	1.01689 (16)	0.0396 (8)	
H21	0.6345	0.3577	1.0218	0.048*	
C22	0.5392 (2)	0.28906 (18)	0.98522 (14)	0.0317 (7)	
C23	0.4766 (2)	0.34208 (17)	0.95560 (15)	0.0317 (7)	
C24	0.4995 (2)	0.41366 (19)	0.94928 (17)	0.0386 (8)	
H24	0.5548	0.4282	0.9663	0.046*	
C25	0.4432 (3)	0.4633 (2)	0.91884 (18)	0.0428 (9)	
H25	0.4599	0.5108	0.9166	0.051*	
C26	0.3618 (2)	0.44223 (19)	0.89172 (17)	0.0408 (8)	
H26	0.3242	0.4751	0.8696	0.049*	
C27	0.3360 (2)	0.37214 (18)	0.89741 (16)	0.0363 (8)	
H27	0.2809	0.3585	0.8790	0.044*	
C28	0.3911 (2)	0.32129 (17)	0.93032 (15)	0.0312 (7)	
C29	0.0764 (2)	0.10264 (17)	0.76379 (14)	0.0291 (7)	
C30	0.1064 (2)	0.03055 (17)	0.75287 (15)	0.0309 (7)	
C31	0.0435 (2)	−0.02258 (18)	0.72335 (15)	0.0346 (7)	
C32	0.0685 (2)	−0.0803 (2)	0.69200 (18)	0.0429 (9)	
H32	0.1254	−0.0851	0.6889	0.051*	
C33	0.0088 (3)	−0.1305 (2)	0.6655 (2)	0.0529 (10)	
H33	0.0255	−0.1689	0.6445	0.064*	
C34	−0.0749 (3)	−0.1236 (2)	0.6701 (2)	0.0539 (11)	
H34	−0.1146	−0.1579	0.6526	0.065*	
C35	−0.1009 (2)	−0.0668 (2)	0.70016 (19)	0.0472 (9)	
H35	−0.1581	−0.0628	0.7026	0.057*	
C36	−0.0422 (2)	−0.01423 (19)	0.72739 (16)	0.0366 (8)	
C37	−0.0676 (2)	0.0483 (2)	0.75883 (16)	0.0360 (8)	
C38	−0.1479 (2)	0.0511 (2)	0.77467 (18)	0.0434 (9)	
H38	−0.1845	0.0120	0.7661	0.052*	
C39	−0.1741 (2)	0.1100 (2)	0.80252 (18)	0.0462 (9)	
H39	−0.2275	0.1102	0.8127	0.055*	
C40	−0.1214 (2)	0.1683 (2)	0.81519 (18)	0.0450 (9)	
H40	−0.1397	0.2086	0.8331	0.054*	
C41	−0.0410 (2)	0.1676 (2)	0.80142 (17)	0.0393 (8)	
H41	−0.0054	0.2072	0.8109	0.047*	
C42	−0.0122 (2)	0.10822 (18)	0.77336 (15)	0.0323 (7)	
C43	0.0836 (2)	0.08681 (19)	0.92655 (15)	0.0345 (7)	
C44	0.1243 (2)	0.02254 (19)	0.90908 (15)	0.0346 (7)	
C45	0.0739 (2)	−0.04203 (19)	0.89031 (16)	0.0378 (8)	
C46	0.1065 (3)	−0.1008 (2)	0.86405 (17)	0.0433 (9)	
H46	0.1639	−0.1016	0.8616	0.052*	
C47	0.0542 (3)	−0.1577 (2)	0.8417 (2)	0.0555 (11)	
H47	0.0760	−0.1960	0.8233	0.067*	
C48	−0.0306 (3)	−0.1576 (3)	0.8468 (2)	0.0633 (13)	
H48	−0.0655	−0.1963	0.8323	0.076*	
C49	−0.0636 (3)	−0.1003 (2)	0.8731 (2)	0.0535 (11)	
H49	−0.1209	−0.1011	0.8758	0.064*	
C50	−0.0134 (2)	−0.0412 (2)	0.89578 (16)	0.0424 (9)	
C51	−0.0494 (2)	0.0194 (2)	0.92335 (16)	0.0408 (9)	
C52	−0.1321 (3)	0.0157 (2)	0.93653 (19)	0.0505 (10)	
H52	−0.1643	−0.0258	0.9266	0.061*	
C53	−0.1662 (2)	0.0706 (3)	0.96312 (19)	0.0524 (11)	
H53	−0.2208	0.0661	0.9712	0.063*	
C54	−0.1207 (2)	0.1331 (3)	0.97825 (18)	0.0507 (10)	
H54	−0.1443	0.1706	0.9964	0.061*	
C55	−0.0392 (2)	0.1395 (2)	0.96614 (17)	0.0430 (9)	
H55	−0.0084	0.1816	0.9762	0.052*	
C56	−0.0028 (2)	0.0833 (2)	0.93893 (15)	0.0379 (8)	
C57	0.4778 (2)	−0.09599 (17)	0.87975 (15)	0.0311 (7)	
C58	0.4046 (2)	−0.13101 (17)	0.83829 (15)	0.0305 (7)	
C59	0.4055 (2)	−0.20914 (17)	0.83084 (14)	0.0320 (7)	
C60	0.3314 (2)	−0.24949 (19)	0.80879 (17)	0.0412 (8)	
H60	0.2797	−0.2260	0.7955	0.049*	
C61	0.3342 (3)	−0.3230 (2)	0.80656 (19)	0.0508 (10)	
H61	0.2842	−0.3488	0.7931	0.061*	
C62	0.4101 (3)	−0.3585 (2)	0.82407 (19)	0.0534 (11)	
H62	0.4120	−0.4082	0.8214	0.064*	
C63	0.4839 (3)	−0.3203 (2)	0.84575 (19)	0.0487 (10)	
H63	0.5352	−0.3449	0.8576	0.058*	
C64	0.4835 (2)	−0.24541 (18)	0.85028 (16)	0.0367 (8)	
C65	0.5628 (2)	−0.20611 (19)	0.87678 (16)	0.0367 (8)	
C66	0.6421 (2)	−0.2409 (2)	0.88592 (18)	0.0453 (9)	
H66	0.6448	−0.2878	0.8724	0.054*	
C67	0.7154 (3)	−0.2078 (2)	0.91426 (19)	0.0483 (10)	
H67	0.7673	−0.2321	0.9199	0.058*	
C68	0.7123 (2)	−0.1384 (2)	0.93455 (18)	0.0441 (9)	
H68	0.7622	−0.1167	0.9550	0.053*	
C69	0.6367 (2)	−0.10049 (19)	0.92512 (16)	0.0372 (8)	
H69	0.6360	−0.0534	0.9384	0.045*	
C70	0.5598 (2)	−0.13368 (18)	0.89503 (15)	0.0321 (7)	
C71	0.1134 (3)	0.1368 (4)	0.5502 (3)	0.100 (2)	
H71A	0.0755	0.1011	0.5291	0.150*	
H71B	0.1188	0.1755	0.5235	0.150*	
H71C	0.0906	0.1547	0.5832	0.150*	
C72	0.2006 (3)	0.1037 (3)	0.5736 (2)	0.0596 (12)	
C73	0.2598 (3)	0.0972 (2)	0.5371 (2)	0.0513 (10)	
H73	0.2466	0.1145	0.4980	0.062*	
C74	0.3375 (3)	0.0656 (2)	0.55792 (19)	0.0501 (10)	
H74	0.3767	0.0618	0.5330	0.060*	
C75	0.3582 (3)	0.0391 (2)	0.61574 (19)	0.0502 (10)	
H75	0.4106	0.0167	0.6294	0.060*	
C76	0.3013 (3)	0.0460 (3)	0.6527 (2)	0.0634 (13)	
H76	0.3150	0.0287	0.6917	0.076*	
C77	0.2235 (3)	0.0787 (3)	0.6320 (2)	0.0722 (15)	
H77	0.1857	0.0842	0.6578	0.087*	
C78	−0.1860 (5)	0.0902 (4)	0.4357 (4)	0.087 (2)	0.66
H78A	−0.1443	0.0585	0.4248	0.131*	0.66
H78B	−0.1928	0.1314	0.4099	0.131*	0.66
H78C	−0.2397	0.0656	0.4315	0.131*	0.66
C79	−0.1572 (3)	0.1133 (3)	0.4982 (3)	0.0727 (19)	0.66
C80	−0.1792 (5)	0.0748 (4)	0.5449 (3)	0.080 (2)	0.66
H80	−0.2124	0.0336	0.5364	0.096*	0.66
C81	−0.1524 (6)	0.0964 (5)	0.6039 (4)	0.086 (3)	0.66
H81	−0.1681	0.0703	0.6348	0.103*	0.66
C82	−0.1021 (6)	0.1574 (5)	0.6167 (3)	0.082 (3)	0.66
H82	−0.0833	0.1721	0.6562	0.098*	0.66
C83	−0.0800 (5)	0.1963 (4)	0.5703 (3)	0.083 (2)	0.66
H83	−0.0466	0.2373	0.5789	0.100*	0.66
C84	−0.1068 (4)	0.1750 (3)	0.5113 (3)	0.0769 (19)	0.66
H84	−0.0916	0.2015	0.4805	0.092*	0.66
C78A	−0.1035 (9)	0.1587 (8)	0.4495 (6)	0.078 (4)	0.34
H78D	−0.0889	0.2087	0.4523	0.118*	0.34
H78E	−0.1505	0.1513	0.4163	0.118*	0.34
H78F	−0.0551	0.1314	0.4433	0.118*	0.34
C79A	−0.1290 (7)	0.1344 (6)	0.5073 (7)	0.080 (3)	0.34
C81A	−0.2097 (10)	0.0579 (9)	0.5603 (8)	0.082 (3)	0.34
H81A	−0.2488	0.0208	0.5596	0.098*	0.34
C80A	−0.1873 (8)	0.0791 (7)	0.5038 (8)	0.079 (3)	0.34
H80A	−0.2108	0.0567	0.4677	0.094*	0.34
C82A	−0.1790 (13)	0.0872 (12)	0.6100 (11)	0.082 (3)	0.34
H82A	−0.1962	0.0714	0.6444	0.098*	0.34
C83A	−0.1209 (15)	0.1416 (12)	0.6139 (7)	0.082 (3)	0.34
H83A	−0.0979	0.1629	0.6505	0.099*	0.34
C84A	−0.0965 (12)	0.1648 (9)	0.5594 (6)	0.081 (2)	0.34
H84A	−0.0572	0.2019	0.5609	0.097*	0.34
N1	0.27646 (17)	0.24607 (14)	0.76671 (13)	0.0330 (6)	
N2	0.37143 (17)	0.14219 (14)	0.79170 (13)	0.0302 (6)	
N3	0.28561 (17)	0.22388 (14)	0.93075 (12)	0.0312 (6)	
N4	0.42751 (17)	0.12499 (14)	0.93553 (12)	0.0307 (6)	
N5	0.13044 (18)	0.15645 (14)	0.76514 (14)	0.0337 (6)	
N6	0.18439 (17)	0.00730 (14)	0.76635 (13)	0.0308 (6)	
N7	0.13358 (18)	0.14323 (15)	0.93093 (13)	0.0345 (6)	
N8	0.20644 (18)	0.03278 (15)	0.90921 (13)	0.0330 (6)	
N9	0.45806 (17)	−0.03173 (14)	0.89580 (12)	0.0301 (6)	
N10	0.34564 (17)	−0.08566 (14)	0.81453 (13)	0.0329 (6)	
O1	0.21502 (15)	0.29635 (12)	0.75199 (13)	0.0424 (6)	
O2	0.41168 (14)	0.07989 (11)	0.80519 (11)	0.0333 (5)	
O3	0.22535 (15)	0.27495 (13)	0.92507 (12)	0.0399 (6)	
O4	0.34924 (14)	0.09770 (12)	0.91047 (11)	0.0342 (5)	
O5	0.09813 (16)	0.22254 (13)	0.76779 (14)	0.0454 (7)	
O6	0.24766 (14)	0.05433 (12)	0.79020 (11)	0.0331 (5)	
O7	0.10615 (16)	0.20776 (14)	0.94544 (13)	0.0445 (6)	
O8	0.25669 (15)	−0.02059 (12)	0.89833 (11)	0.0361 (5)	
O9	0.52088 (17)	0.00669 (14)	0.93202 (12)	0.0404 (6)	
O10	0.27559 (16)	−0.11284 (13)	0.77446 (12)	0.0395 (6)	
Ni1	0.25135 (3)	0.14989 (2)	0.77620 (2)	0.03005 (11)	
Ni2	0.24612 (3)	0.12803 (2)	0.92204 (2)	0.03014 (11)	
Ni3	0.34675 (3)	0.01380 (2)	0.85238 (2)	0.02978 (11)	
H1A	0.149 (4)	0.262 (3)	0.761 (3)	0.10 (2)*	
H2A	0.166 (3)	0.244 (3)	0.935 (2)	0.081 (16)*	
H3A	0.488 (4)	0.043 (3)	0.933 (3)	0.09 (2)*	
H4A	0.233 (3)	−0.070 (3)	0.770 (2)	0.095 (18)*	

### (1) Bis[µ2-9,10-bis(oxidoimino)phenanthrene]bis[µ2-10-(oxidoimino)phenanthrene-9-one oxime](phenanthrene-9,10-dione dioxime)trinickel(II) toluene disolvate Atomic displacement parameters (Å^2^)

**Table:**

	*U*^11^	*U*^22^	*U*^33^	*U*^12^	*U*^13^	*U*^23^
C1	0.0263 (16)	0.0267 (15)	0.0376 (17)	−0.0007 (13)	0.0076 (13)	0.0027 (13)
C2	0.0271 (16)	0.0272 (15)	0.0329 (16)	0.0010 (13)	0.0088 (13)	0.0019 (13)
C3	0.0274 (16)	0.0301 (16)	0.0318 (16)	−0.0026 (13)	0.0120 (13)	−0.0014 (13)
C4	0.0296 (17)	0.0359 (18)	0.0379 (18)	0.0044 (14)	0.0095 (14)	0.0015 (14)
C5	0.0292 (18)	0.047 (2)	0.0414 (19)	0.0061 (16)	0.0076 (15)	0.0005 (16)
C6	0.0243 (17)	0.055 (2)	0.046 (2)	−0.0013 (16)	0.0035 (15)	−0.0013 (18)
C7	0.0321 (19)	0.045 (2)	0.046 (2)	−0.0098 (16)	0.0084 (15)	−0.0027 (17)
C8	0.0286 (17)	0.0349 (17)	0.0343 (17)	−0.0010 (14)	0.0122 (13)	−0.0019 (14)
C9	0.0319 (17)	0.0291 (16)	0.0334 (16)	−0.0043 (13)	0.0125 (13)	−0.0049 (13)
C10	0.0361 (19)	0.0343 (18)	0.0448 (19)	−0.0093 (15)	0.0173 (15)	−0.0043 (15)
C11	0.049 (2)	0.0278 (17)	0.048 (2)	−0.0082 (16)	0.0199 (17)	0.0011 (15)
C12	0.048 (2)	0.0265 (16)	0.046 (2)	0.0013 (15)	0.0182 (17)	0.0047 (15)
C13	0.0344 (18)	0.0274 (16)	0.0396 (18)	0.0021 (14)	0.0104 (14)	0.0029 (14)
C14	0.0313 (17)	0.0237 (15)	0.0354 (16)	−0.0018 (13)	0.0118 (13)	0.0005 (13)
C15	0.0300 (17)	0.0285 (16)	0.0298 (15)	−0.0013 (13)	0.0080 (13)	−0.0011 (13)
C16	0.0288 (16)	0.0261 (15)	0.0309 (15)	−0.0018 (13)	0.0081 (13)	−0.0004 (12)
C17	0.0282 (16)	0.0318 (16)	0.0285 (15)	−0.0034 (13)	0.0072 (12)	−0.0013 (13)
C18	0.0335 (18)	0.0324 (17)	0.0321 (16)	−0.0039 (14)	0.0062 (14)	−0.0003 (14)
C19	0.0315 (18)	0.043 (2)	0.0338 (17)	0.0031 (15)	0.0052 (14)	0.0020 (15)
C20	0.0312 (18)	0.049 (2)	0.0400 (19)	−0.0079 (16)	−0.0001 (15)	−0.0026 (16)
C21	0.039 (2)	0.0372 (19)	0.0425 (19)	−0.0116 (16)	0.0077 (16)	−0.0027 (16)
C22	0.0322 (17)	0.0329 (17)	0.0312 (16)	−0.0055 (14)	0.0095 (13)	−0.0022 (13)
C23	0.0345 (18)	0.0299 (16)	0.0328 (16)	−0.0010 (14)	0.0116 (14)	−0.0016 (13)
C24	0.040 (2)	0.0316 (17)	0.047 (2)	−0.0064 (15)	0.0132 (16)	−0.0014 (15)
C25	0.053 (2)	0.0287 (17)	0.050 (2)	−0.0040 (16)	0.0183 (18)	−0.0005 (16)
C26	0.048 (2)	0.0298 (17)	0.046 (2)	0.0042 (16)	0.0131 (17)	0.0056 (15)
C27	0.039 (2)	0.0315 (17)	0.0391 (18)	0.0016 (15)	0.0109 (15)	0.0002 (14)
C28	0.0335 (17)	0.0283 (16)	0.0341 (17)	−0.0011 (14)	0.0123 (14)	−0.0011 (13)
C29	0.0243 (16)	0.0294 (16)	0.0328 (16)	0.0010 (13)	0.0043 (12)	0.0051 (13)
C30	0.0267 (16)	0.0299 (16)	0.0355 (17)	−0.0019 (13)	0.0053 (13)	0.0030 (13)
C31	0.0344 (18)	0.0314 (17)	0.0359 (17)	−0.0036 (14)	0.0026 (14)	0.0023 (14)
C32	0.037 (2)	0.041 (2)	0.048 (2)	−0.0034 (16)	0.0033 (16)	−0.0064 (17)
C33	0.050 (3)	0.040 (2)	0.064 (3)	−0.0043 (19)	0.001 (2)	−0.0108 (19)
C34	0.047 (2)	0.042 (2)	0.065 (3)	−0.0139 (19)	−0.006 (2)	−0.003 (2)
C35	0.034 (2)	0.043 (2)	0.060 (2)	−0.0075 (17)	−0.0004 (17)	0.0005 (19)
C36	0.0312 (18)	0.0355 (18)	0.0403 (19)	−0.0055 (14)	0.0007 (14)	0.0057 (15)
C37	0.0289 (17)	0.0405 (19)	0.0374 (18)	−0.0029 (15)	0.0040 (14)	0.0083 (15)
C38	0.0283 (18)	0.052 (2)	0.049 (2)	−0.0044 (16)	0.0051 (16)	0.0110 (18)
C39	0.0297 (19)	0.063 (3)	0.048 (2)	0.0074 (18)	0.0115 (16)	0.0120 (19)
C40	0.033 (2)	0.057 (2)	0.047 (2)	0.0098 (18)	0.0127 (16)	0.0050 (18)
C41	0.0339 (19)	0.0388 (19)	0.046 (2)	0.0010 (15)	0.0088 (15)	−0.0008 (16)
C42	0.0255 (16)	0.0349 (17)	0.0357 (17)	0.0006 (13)	0.0043 (13)	0.0063 (14)
C43	0.0300 (17)	0.0390 (18)	0.0344 (17)	−0.0011 (14)	0.0059 (13)	0.0044 (14)
C44	0.0328 (18)	0.0368 (18)	0.0328 (17)	−0.0041 (14)	0.0035 (14)	0.0078 (14)
C45	0.038 (2)	0.0362 (18)	0.0372 (18)	−0.0071 (15)	0.0031 (15)	0.0051 (15)
C46	0.043 (2)	0.041 (2)	0.044 (2)	−0.0107 (17)	0.0031 (16)	0.0024 (16)
C47	0.063 (3)	0.044 (2)	0.058 (3)	−0.017 (2)	0.008 (2)	−0.005 (2)
C48	0.056 (3)	0.058 (3)	0.071 (3)	−0.030 (2)	0.001 (2)	−0.004 (2)
C49	0.043 (2)	0.054 (3)	0.060 (3)	−0.017 (2)	0.0017 (19)	0.002 (2)
C50	0.038 (2)	0.047 (2)	0.0387 (19)	−0.0151 (17)	0.0003 (15)	0.0102 (17)
C51	0.0311 (19)	0.053 (2)	0.0361 (18)	−0.0055 (16)	0.0019 (14)	0.0130 (16)
C52	0.037 (2)	0.064 (3)	0.049 (2)	−0.0115 (19)	0.0057 (17)	0.016 (2)
C53	0.030 (2)	0.078 (3)	0.051 (2)	0.002 (2)	0.0115 (17)	0.019 (2)
C54	0.035 (2)	0.074 (3)	0.046 (2)	0.005 (2)	0.0135 (17)	0.011 (2)
C55	0.0318 (19)	0.054 (2)	0.045 (2)	0.0010 (17)	0.0108 (16)	0.0073 (18)
C56	0.0281 (17)	0.050 (2)	0.0341 (17)	−0.0037 (16)	0.0025 (14)	0.0097 (16)
C57	0.0320 (17)	0.0263 (15)	0.0364 (17)	0.0007 (13)	0.0102 (14)	0.0033 (13)
C58	0.0323 (17)	0.0262 (15)	0.0341 (16)	0.0016 (13)	0.0095 (13)	0.0031 (13)
C59	0.0381 (18)	0.0273 (16)	0.0303 (16)	0.0003 (14)	0.0061 (14)	0.0001 (13)
C60	0.042 (2)	0.0313 (18)	0.048 (2)	−0.0002 (15)	0.0036 (16)	0.0007 (16)
C61	0.065 (3)	0.0309 (19)	0.050 (2)	−0.0080 (19)	−0.003 (2)	−0.0026 (17)
C62	0.076 (3)	0.0271 (18)	0.053 (2)	0.0001 (19)	0.003 (2)	−0.0054 (17)
C63	0.058 (3)	0.0330 (19)	0.053 (2)	0.0136 (18)	0.0061 (19)	0.0004 (17)
C64	0.047 (2)	0.0299 (17)	0.0355 (18)	0.0032 (15)	0.0124 (15)	0.0008 (14)
C65	0.0396 (19)	0.0345 (18)	0.0382 (18)	0.0070 (15)	0.0130 (15)	0.0062 (15)
C66	0.045 (2)	0.039 (2)	0.054 (2)	0.0106 (17)	0.0135 (18)	0.0073 (17)
C67	0.038 (2)	0.047 (2)	0.060 (2)	0.0133 (18)	0.0112 (18)	0.0134 (19)
C68	0.0332 (19)	0.047 (2)	0.050 (2)	0.0003 (16)	0.0055 (16)	0.0147 (18)
C69	0.0358 (19)	0.0345 (18)	0.0411 (19)	0.0052 (15)	0.0074 (15)	0.0093 (15)
C70	0.0312 (17)	0.0303 (16)	0.0356 (17)	0.0021 (14)	0.0085 (14)	0.0062 (14)
C71	0.055 (3)	0.108 (5)	0.138 (6)	0.015 (3)	0.025 (4)	0.052 (4)
C72	0.047 (3)	0.054 (3)	0.080 (3)	0.000 (2)	0.019 (2)	0.015 (2)
C73	0.055 (3)	0.052 (2)	0.050 (2)	−0.001 (2)	0.0157 (19)	0.0116 (19)
C74	0.059 (3)	0.045 (2)	0.053 (2)	0.000 (2)	0.026 (2)	−0.0001 (19)
C75	0.054 (3)	0.045 (2)	0.053 (2)	0.0034 (19)	0.014 (2)	0.0046 (19)
C76	0.070 (3)	0.074 (3)	0.052 (3)	0.018 (3)	0.025 (2)	0.016 (2)
C77	0.075 (3)	0.083 (4)	0.071 (3)	0.017 (3)	0.045 (3)	0.020 (3)
C78	0.110 (6)	0.077 (5)	0.074 (5)	−0.002 (5)	0.018 (5)	−0.001 (4)
C79	0.081 (4)	0.068 (4)	0.068 (3)	0.006 (4)	0.014 (3)	−0.012 (3)
C80	0.100 (5)	0.069 (4)	0.067 (4)	0.005 (4)	0.006 (4)	−0.005 (3)
C81	0.105 (5)	0.069 (4)	0.075 (4)	0.007 (4)	−0.001 (4)	0.004 (4)
C82	0.097 (5)	0.070 (5)	0.069 (4)	0.009 (4)	−0.005 (4)	−0.009 (3)
C83	0.088 (4)	0.078 (5)	0.080 (4)	0.007 (4)	0.007 (4)	−0.015 (4)
C84	0.080 (4)	0.075 (4)	0.076 (4)	0.010 (4)	0.018 (4)	−0.010 (4)
C78A	0.087 (8)	0.089 (8)	0.067 (7)	0.002 (7)	0.035 (6)	0.000 (7)
C79A	0.090 (5)	0.074 (5)	0.074 (4)	0.010 (4)	0.015 (4)	−0.008 (4)
C81A	0.098 (6)	0.074 (6)	0.070 (5)	0.002 (5)	0.010 (5)	−0.003 (5)
C80A	0.093 (6)	0.070 (5)	0.072 (5)	0.001 (5)	0.014 (5)	−0.005 (5)
C82A	0.097 (6)	0.072 (5)	0.071 (5)	0.007 (5)	0.004 (5)	0.000 (4)
C83A	0.095 (6)	0.074 (5)	0.071 (4)	0.006 (5)	−0.003 (5)	−0.005 (5)
C84A	0.090 (5)	0.074 (5)	0.075 (4)	0.008 (4)	0.007 (4)	−0.009 (4)
N1	0.0260 (14)	0.0272 (14)	0.0453 (16)	0.0056 (11)	0.0061 (12)	0.0058 (12)
N2	0.0292 (14)	0.0235 (13)	0.0386 (15)	0.0031 (11)	0.0087 (12)	0.0020 (11)
N3	0.0283 (14)	0.0289 (14)	0.0366 (15)	0.0044 (11)	0.0072 (11)	0.0010 (11)
N4	0.0267 (14)	0.0288 (13)	0.0366 (14)	−0.0035 (11)	0.0064 (11)	−0.0041 (11)
N5	0.0284 (15)	0.0259 (13)	0.0478 (17)	0.0024 (11)	0.0104 (12)	0.0030 (12)
N6	0.0275 (14)	0.0251 (13)	0.0394 (15)	−0.0040 (11)	0.0060 (12)	0.0006 (11)
N7	0.0304 (15)	0.0345 (15)	0.0379 (15)	−0.0016 (12)	0.0058 (12)	0.0002 (12)
N8	0.0307 (15)	0.0301 (14)	0.0389 (15)	−0.0007 (12)	0.0084 (12)	0.0027 (12)
N9	0.0279 (14)	0.0247 (13)	0.0365 (14)	−0.0019 (11)	0.0034 (11)	0.0023 (11)
N10	0.0294 (15)	0.0264 (13)	0.0415 (16)	−0.0013 (11)	0.0039 (12)	−0.0017 (12)
O1	0.0271 (13)	0.0255 (12)	0.0740 (19)	0.0064 (10)	0.0091 (12)	0.0141 (12)
O2	0.0295 (12)	0.0231 (11)	0.0480 (14)	0.0047 (9)	0.0098 (10)	0.0017 (10)
O3	0.0292 (13)	0.0282 (12)	0.0627 (17)	0.0058 (10)	0.0104 (11)	0.0020 (11)
O4	0.0255 (12)	0.0296 (12)	0.0474 (14)	−0.0045 (9)	0.0072 (10)	−0.0069 (10)
O5	0.0288 (13)	0.0255 (12)	0.084 (2)	0.0048 (10)	0.0163 (13)	0.0077 (13)
O6	0.0249 (12)	0.0255 (11)	0.0468 (14)	−0.0039 (9)	0.0021 (10)	0.0009 (10)
O7	0.0333 (14)	0.0368 (14)	0.0663 (18)	0.0036 (11)	0.0169 (12)	−0.0043 (13)
O8	0.0344 (13)	0.0292 (12)	0.0449 (14)	−0.0010 (10)	0.0087 (11)	0.0005 (10)
O9	0.0337 (14)	0.0317 (13)	0.0506 (15)	−0.0002 (11)	−0.0038 (11)	−0.0058 (11)
O10	0.0349 (14)	0.0309 (12)	0.0471 (15)	0.0019 (11)	−0.0049 (11)	−0.0071 (11)
Ni1	0.0237 (2)	0.0232 (2)	0.0434 (2)	0.00088 (16)	0.00702 (17)	0.00455 (17)
Ni2	0.0251 (2)	0.0275 (2)	0.0382 (2)	−0.00170 (16)	0.00718 (17)	−0.00033 (17)
Ni3	0.0274 (2)	0.0223 (2)	0.0386 (2)	0.00040 (16)	0.00435 (17)	0.00011 (17)

### (1) Bis[µ2-9,10-bis(oxidoimino)phenanthrene]bis[µ2-10-(oxidoimino)phenanthrene-9-one oxime](phenanthrene-9,10-dione dioxime)trinickel(II) toluene disolvate Geometric parameters (Å, º)

**Table:**

C1—N1	1.310 (4)	C55—H55	0.9300
C1—C14	1.461 (4)	C57—N9	1.310 (4)
C1—C2	1.464 (4)	C57—C70	1.465 (5)
C2—N2	1.322 (4)	C57—C58	1.495 (5)
C2—C3	1.462 (4)	C58—N10	1.299 (4)
C3—C4	1.410 (5)	C58—C59	1.467 (4)
C3—C8	1.418 (4)	C59—C60	1.406 (5)
C4—C5	1.373 (5)	C59—C64	1.408 (5)
C4—H4	0.9300	C60—C61	1.372 (5)
C5—C6	1.383 (5)	C60—H60	0.9300
C5—H5	0.9300	C61—C62	1.368 (6)
C6—C7	1.374 (5)	C61—H61	0.9300
C6—H6	0.9300	C62—C63	1.380 (6)
C7—C8	1.400 (5)	C62—H62	0.9300
C7—H7	0.9300	C63—C64	1.399 (5)
C8—C9	1.479 (5)	C63—H63	0.9300
C9—C10	1.406 (4)	C64—C65	1.481 (5)
C9—C14	1.412 (5)	C65—C66	1.400 (5)
C10—C11	1.373 (5)	C65—C70	1.416 (5)
C10—H10	0.9300	C66—C67	1.364 (6)
C11—C12	1.387 (5)	C66—H66	0.9300
C11—H11	0.9300	C67—C68	1.379 (6)
C12—C13	1.384 (5)	C67—H67	0.9300
C12—H12	0.9300	C68—C69	1.378 (5)
C13—C14	1.408 (4)	C68—H68	0.9300
C13—H13	0.9300	C69—C70	1.420 (5)
C15—N3	1.321 (4)	C69—H69	0.9300
C15—C16	1.468 (4)	C71—C72	1.517 (7)
C15—C28	1.479 (4)	C71—H71A	0.9600
C16—N4	1.290 (4)	C71—H71B	0.9600
C16—C17	1.481 (4)	C71—H71C	0.9600
C17—C22	1.396 (4)	C72—C73	1.387 (6)
C17—C18	1.401 (5)	C72—C77	1.388 (7)
C18—C19	1.380 (5)	C73—C74	1.369 (6)
C18—H18	0.9300	C73—H73	0.9300
C19—C20	1.390 (5)	C74—C75	1.382 (6)
C19—H19	0.9300	C74—H74	0.9300
C20—C21	1.372 (5)	C75—C76	1.365 (6)
C20—H20	0.9300	C75—H75	0.9300
C21—C22	1.410 (5)	C76—C77	1.377 (7)
C21—H21	0.9300	C76—H76	0.9300
C22—C23	1.469 (5)	C77—H77	0.9300
C23—C24	1.399 (5)	C78—C79	1.470 (10)
C23—C28	1.424 (5)	C78—H78A	0.9600
C24—C25	1.376 (5)	C78—H78B	0.9600
C24—H24	0.9300	C78—H78C	0.9600
C25—C26	1.378 (5)	C79—C80	1.386 (6)
C25—H25	0.9300	C79—C84	1.401 (6)
C26—C27	1.384 (5)	C80—C81	1.386 (7)
C26—H26	0.9300	C80—H80	0.9300
C27—C28	1.402 (5)	C81—C82	1.390 (7)
C27—H27	0.9300	C81—H81	0.9300
C29—N5	1.319 (4)	C82—C83	1.386 (7)
C29—C30	1.465 (4)	C82—H82	0.9300
C29—C42	1.479 (4)	C83—C84	1.384 (6)
C30—N6	1.295 (4)	C83—H83	0.9300
C30—C31	1.472 (5)	C84—H84	0.9300
C31—C32	1.394 (5)	C78A—C79A	1.523 (18)
C31—C36	1.399 (5)	C78A—H78D	0.9600
C32—C33	1.384 (5)	C78A—H78E	0.9600
C32—H32	0.9300	C78A—H78F	0.9600
C33—C34	1.367 (6)	C79A—C84A	1.32 (2)
C33—H33	0.9300	C79A—C80A	1.382 (8)
C34—C35	1.372 (6)	C81A—C82A	1.26 (3)
C34—H34	0.9300	C81A—C80A	1.46 (2)
C35—C36	1.410 (5)	C81A—H81A	0.9300
C35—H35	0.9300	C80A—H80A	0.9300
C36—C37	1.469 (5)	C82A—C83A	1.36 (3)
C37—C38	1.403 (5)	C82A—H82A	0.9300
C37—C42	1.420 (5)	C83A—C84A	1.44 (2)
C38—C39	1.375 (6)	C83A—H83A	0.9300
C38—H38	0.9300	C84A—H84A	0.9300
C39—C40	1.370 (6)	N1—O1	1.349 (3)
C39—H39	0.9300	N1—Ni1	1.859 (3)
C40—C41	1.382 (5)	N2—O2	1.333 (3)
C40—H40	0.9300	N2—Ni1	1.884 (3)
C41—C42	1.401 (5)	N3—O3	1.341 (3)
C41—H41	0.9300	N3—Ni2	1.892 (3)
C43—N7	1.311 (4)	N4—O4	1.363 (3)
C43—C44	1.457 (5)	N5—O5	1.342 (3)
C43—C56	1.465 (5)	N5—Ni1	1.900 (3)
C44—N8	1.325 (4)	N6—O6	1.364 (3)
C44—C45	1.463 (5)	N7—O7	1.344 (4)
C45—C46	1.399 (5)	N7—Ni2	1.871 (3)
C45—C50	1.425 (5)	N8—O8	1.333 (4)
C46—C47	1.381 (5)	N8—Ni2	1.888 (3)
C46—H46	0.9300	N9—O9	1.365 (4)
C47—C48	1.383 (6)	N9—Ni3	2.035 (3)
C47—H47	0.9300	N10—O10	1.388 (4)
C48—C49	1.381 (7)	N10—Ni3	2.043 (3)
C48—H48	0.9300	O1—H1A	1.29 (6)
C49—C50	1.399 (5)	O2—Ni3	2.050 (2)
C49—H49	0.9300	O3—H2A	1.17 (5)
C50—C51	1.465 (6)	O4—Ni2	1.811 (2)
C51—C56	1.411 (5)	O4—Ni3	2.044 (2)
C51—C52	1.416 (5)	O5—H1A	1.13 (6)
C52—C53	1.359 (6)	O6—Ni1	1.813 (2)
C52—H52	0.9300	O6—Ni3	2.042 (2)
C53—C54	1.378 (6)	O7—H2A	1.24 (6)
C53—H53	0.9300	O8—Ni3	2.047 (2)
C54—C55	1.389 (5)	O9—H3A	0.85 (6)
C54—H54	0.9300	O10—H4A	1.04 (6)
C55—C56	1.402 (5)		
			
N1—C1—C14	127.3 (3)	C60—C59—C58	123.0 (3)
N1—C1—C2	112.3 (3)	C64—C59—C58	118.0 (3)
C14—C1—C2	120.4 (3)	C61—C60—C59	121.1 (4)
N2—C2—C3	127.7 (3)	C61—C60—H60	119.5
N2—C2—C1	111.7 (3)	C59—C60—H60	119.5
C3—C2—C1	120.3 (3)	C62—C61—C60	120.4 (4)
C4—C3—C8	119.3 (3)	C62—C61—H61	119.8
C4—C3—C2	122.9 (3)	C60—C61—H61	119.8
C8—C3—C2	117.5 (3)	C61—C62—C63	119.8 (4)
C5—C4—C3	120.8 (3)	C61—C62—H62	120.1
C5—C4—H4	119.6	C63—C62—H62	120.1
C3—C4—H4	119.6	C62—C63—C64	121.7 (4)
C4—C5—C6	120.1 (3)	C62—C63—H63	119.2
C4—C5—H5	120.0	C64—C63—H63	119.2
C6—C5—H5	120.0	C63—C64—C59	118.2 (3)
C7—C6—C5	120.0 (3)	C63—C64—C65	120.5 (3)
C7—C6—H6	120.0	C59—C64—C65	121.3 (3)
C5—C6—H6	120.0	C66—C65—C70	118.8 (3)
C6—C7—C8	122.1 (4)	C66—C65—C64	120.2 (3)
C6—C7—H7	119.0	C70—C65—C64	120.9 (3)
C8—C7—H7	119.0	C67—C66—C65	121.6 (4)
C7—C8—C3	117.6 (3)	C67—C66—H66	119.2
C7—C8—C9	121.2 (3)	C65—C66—H66	119.2
C3—C8—C9	121.2 (3)	C66—C67—C68	119.8 (4)
C10—C9—C14	117.7 (3)	C66—C67—H67	120.1
C10—C9—C8	121.0 (3)	C68—C67—H67	120.1
C14—C9—C8	121.3 (3)	C69—C68—C67	121.3 (4)
C11—C10—C9	122.2 (3)	C69—C68—H68	119.3
C11—C10—H10	118.9	C67—C68—H68	119.3
C9—C10—H10	118.9	C68—C69—C70	119.7 (3)
C10—C11—C12	120.0 (3)	C68—C69—H69	120.1
C10—C11—H11	120.0	C70—C69—H69	120.1
C12—C11—H11	120.0	C65—C70—C69	118.7 (3)
C13—C12—C11	119.5 (3)	C65—C70—C57	118.0 (3)
C13—C12—H12	120.2	C69—C70—C57	123.3 (3)
C11—C12—H12	120.2	C72—C71—H71A	109.5
C12—C13—C14	121.1 (3)	C72—C71—H71B	109.5
C12—C13—H13	119.4	H71A—C71—H71B	109.5
C14—C13—H13	119.4	C72—C71—H71C	109.5
C13—C14—C9	119.4 (3)	H71A—C71—H71C	109.5
C13—C14—C1	122.9 (3)	H71B—C71—H71C	109.5
C9—C14—C1	117.6 (3)	C73—C72—C77	117.9 (4)
N3—C15—C16	118.6 (3)	C73—C72—C71	121.0 (5)
N3—C15—C28	124.4 (3)	C77—C72—C71	121.2 (5)
C16—C15—C28	117.0 (3)	C74—C73—C72	120.7 (4)
N4—C16—C15	126.2 (3)	C74—C73—H73	119.7
N4—C16—C17	115.3 (3)	C72—C73—H73	119.7
C15—C16—C17	118.5 (3)	C73—C74—C75	120.6 (4)
C22—C17—C18	120.3 (3)	C73—C74—H74	119.7
C22—C17—C16	119.6 (3)	C75—C74—H74	119.7
C18—C17—C16	120.1 (3)	C76—C75—C74	119.6 (4)
C19—C18—C17	120.7 (3)	C76—C75—H75	120.2
C19—C18—H18	119.6	C74—C75—H75	120.2
C17—C18—H18	119.6	C75—C76—C77	119.9 (4)
C18—C19—C20	119.5 (3)	C75—C76—H76	120.0
C18—C19—H19	120.3	C77—C76—H76	120.0
C20—C19—H19	120.3	C76—C77—C72	121.3 (4)
C21—C20—C19	120.2 (3)	C76—C77—H77	119.3
C21—C20—H20	119.9	C72—C77—H77	119.3
C19—C20—H20	119.9	C79—C78—H78A	109.5
C20—C21—C22	121.7 (3)	C79—C78—H78B	109.5
C20—C21—H21	119.2	H78A—C78—H78B	109.5
C22—C21—H21	119.2	C79—C78—H78C	109.5
C17—C22—C21	117.7 (3)	H78A—C78—H78C	109.5
C17—C22—C23	120.3 (3)	H78B—C78—H78C	109.5
C21—C22—C23	122.0 (3)	C80—C79—C84	119.1 (5)
C24—C23—C28	117.8 (3)	C80—C79—C78	121.0 (5)
C24—C23—C22	121.4 (3)	C84—C79—C78	119.9 (5)
C28—C23—C22	120.7 (3)	C81—C80—C79	121.2 (5)
C25—C24—C23	122.4 (3)	C81—C80—H80	119.4
C25—C24—H24	118.8	C79—C80—H80	119.4
C23—C24—H24	118.8	C80—C81—C82	119.5 (5)
C24—C25—C26	119.6 (3)	C80—C81—H81	120.2
C24—C25—H25	120.2	C82—C81—H81	120.2
C26—C25—H25	120.2	C83—C82—C81	119.6 (5)
C25—C26—C27	119.9 (4)	C83—C82—H82	120.2
C25—C26—H26	120.0	C81—C82—H82	120.2
C27—C26—H26	120.0	C84—C83—C82	121.0 (5)
C26—C27—C28	121.5 (3)	C84—C83—H83	119.5
C26—C27—H27	119.3	C82—C83—H83	119.5
C28—C27—H27	119.3	C83—C84—C79	119.5 (5)
C27—C28—C23	118.6 (3)	C83—C84—H84	120.2
C27—C28—C15	122.4 (3)	C79—C84—H84	120.2
C23—C28—C15	118.9 (3)	C79A—C78A—H78D	109.5
N5—C29—C30	117.8 (3)	C79A—C78A—H78E	109.5
N5—C29—C42	125.7 (3)	H78D—C78A—H78E	109.5
C30—C29—C42	116.4 (3)	C79A—C78A—H78F	109.5
N6—C30—C29	126.8 (3)	H78D—C78A—H78F	109.5
N6—C30—C31	114.6 (3)	H78E—C78A—H78F	109.5
C29—C30—C31	118.6 (3)	C84A—C79A—C80A	120.4 (17)
C32—C31—C36	120.4 (3)	C84A—C79A—C78A	121.6 (11)
C32—C31—C30	120.9 (3)	C80A—C79A—C78A	117.9 (12)
C36—C31—C30	118.7 (3)	C82A—C81A—C80A	123.7 (16)
C33—C32—C31	120.1 (4)	C82A—C81A—H81A	118.2
C33—C32—H32	120.0	C80A—C81A—H81A	118.2
C31—C32—H32	120.0	C79A—C80A—C81A	115.9 (14)
C34—C33—C32	120.1 (4)	C79A—C80A—H80A	122.1
C34—C33—H33	119.9	C81A—C80A—H80A	122.1
C32—C33—H33	119.9	C81A—C82A—C83A	121 (2)
C33—C34—C35	120.7 (4)	C81A—C82A—H82A	119.5
C33—C34—H34	119.7	C83A—C82A—H82A	119.5
C35—C34—H34	119.7	C82A—C83A—C84A	117.8 (17)
C34—C35—C36	121.0 (4)	C82A—C83A—H83A	121.1
C34—C35—H35	119.5	C84A—C83A—H83A	121.1
C36—C35—H35	119.5	C79A—C84A—C83A	121.3 (18)
C31—C36—C35	117.8 (3)	C79A—C84A—H84A	119.3
C31—C36—C37	119.5 (3)	C83A—C84A—H84A	119.3
C35—C36—C37	122.8 (3)	C1—N1—O1	120.2 (3)
C38—C37—C42	118.0 (3)	C1—N1—Ni1	117.4 (2)
C38—C37—C36	120.8 (3)	O1—N1—Ni1	122.4 (2)
C42—C37—C36	121.1 (3)	C2—N2—O2	121.3 (3)
C39—C38—C37	121.9 (4)	C2—N2—Ni1	116.3 (2)
C39—C38—H38	119.1	O2—N2—Ni1	122.3 (2)
C37—C38—H38	119.1	C15—N3—O3	116.8 (3)
C40—C39—C38	120.0 (4)	C15—N3—Ni2	126.6 (2)
C40—C39—H39	120.0	O3—N3—Ni2	116.3 (2)
C38—C39—H39	120.0	C16—N4—O4	119.1 (3)
C39—C40—C41	120.2 (4)	C29—N5—O5	116.3 (3)
C39—C40—H40	119.9	C29—N5—Ni1	126.6 (2)
C41—C40—H40	119.9	O5—N5—Ni1	116.3 (2)
C40—C41—C42	121.2 (4)	C30—N6—O6	118.6 (3)
C40—C41—H41	119.4	C43—N7—O7	120.8 (3)
C42—C41—H41	119.4	C43—N7—Ni2	117.0 (2)
C41—C42—C37	118.7 (3)	O7—N7—Ni2	122.0 (2)
C41—C42—C29	122.4 (3)	C44—N8—O8	121.7 (3)
C37—C42—C29	118.6 (3)	C44—N8—Ni2	115.9 (2)
N7—C43—C44	112.4 (3)	O8—N8—Ni2	122.3 (2)
N7—C43—C56	127.1 (3)	C57—N9—O9	117.4 (3)
C44—C43—C56	120.4 (3)	C57—N9—Ni3	118.8 (2)
N8—C44—C43	112.2 (3)	O9—N9—Ni3	122.5 (2)
N8—C44—C45	127.3 (3)	C58—N10—O10	117.1 (3)
C43—C44—C45	120.4 (3)	C58—N10—Ni3	118.1 (2)
C46—C45—C50	120.2 (3)	O10—N10—Ni3	123.1 (2)
C46—C45—C44	122.5 (3)	N1—O1—H1A	101 (3)
C50—C45—C44	117.1 (3)	N2—O2—Ni3	111.88 (18)
C47—C46—C45	120.6 (4)	N3—O3—H2A	103 (2)
C47—C46—H46	119.7	N4—O4—Ni2	127.34 (19)
C45—C46—H46	119.7	N4—O4—Ni3	116.88 (18)
C46—C47—C48	119.8 (4)	Ni2—O4—Ni3	115.78 (11)
C46—C47—H47	120.1	N5—O5—H1A	107 (3)
C48—C47—H47	120.1	N6—O6—Ni1	127.55 (19)
C49—C48—C47	120.4 (4)	N6—O6—Ni3	116.43 (18)
C49—C48—H48	119.8	Ni1—O6—Ni3	115.93 (11)
C47—C48—H48	119.8	N7—O7—H2A	98 (2)
C48—C49—C50	121.9 (4)	N8—O8—Ni3	111.97 (18)
C48—C49—H49	119.0	N9—O9—H3A	93 (4)
C50—C49—H49	119.0	N10—O10—H4A	102 (3)
C49—C50—C45	117.1 (4)	O6—Ni1—N1	169.58 (11)
C49—C50—C51	121.4 (4)	O6—Ni1—N2	87.73 (10)
C45—C50—C51	121.5 (3)	N1—Ni1—N2	81.94 (11)
C56—C51—C52	117.1 (4)	O6—Ni1—N5	91.03 (11)
C56—C51—C50	121.7 (3)	N1—Ni1—N5	99.21 (12)
C52—C51—C50	121.2 (4)	N2—Ni1—N5	176.81 (12)
C53—C52—C51	122.3 (4)	O4—Ni2—N7	170.22 (11)
C53—C52—H52	118.9	O4—Ni2—N8	88.31 (11)
C51—C52—H52	118.9	N7—Ni2—N8	81.93 (12)
C52—C53—C54	120.6 (4)	O4—Ni2—N3	90.94 (11)
C52—C53—H53	119.7	N7—Ni2—N3	98.79 (12)
C54—C53—H53	119.7	N8—Ni2—N3	177.16 (12)
C53—C54—C55	119.4 (4)	N9—Ni3—O6	165.53 (11)
C53—C54—H54	120.3	N9—Ni3—N10	76.02 (11)
C55—C54—H54	120.3	O6—Ni3—N10	95.97 (10)
C54—C55—C56	120.9 (4)	N9—Ni3—O4	96.00 (10)
C54—C55—H55	119.5	O6—Ni3—O4	94.37 (10)
C56—C55—H55	119.5	N10—Ni3—O4	164.78 (11)
C55—C56—C51	119.8 (3)	N9—Ni3—O8	104.85 (10)
C55—C56—C43	123.0 (3)	O6—Ni3—O8	86.82 (10)
C51—C56—C43	117.3 (3)	N10—Ni3—O8	89.16 (10)
N9—C57—C70	128.8 (3)	O4—Ni3—O8	80.29 (9)
N9—C57—C58	112.0 (3)	N9—Ni3—O2	91.16 (10)
C70—C57—C58	119.2 (3)	O6—Ni3—O2	79.47 (9)
N10—C58—C59	128.4 (3)	N10—Ni3—O2	106.60 (10)
N10—C58—C57	112.7 (3)	O4—Ni3—O2	86.25 (9)
C59—C58—C57	118.8 (3)	O8—Ni3—O2	159.99 (9)
C60—C59—C64	118.8 (3)		
			
N1—C1—C2—N2	6.8 (4)	C50—C51—C56—C43	−0.1 (5)
C14—C1—C2—N2	−174.0 (3)	N7—C43—C56—C55	−9.7 (6)
N1—C1—C2—C3	−166.9 (3)	C44—C43—C56—C55	168.1 (3)
C14—C1—C2—C3	12.4 (5)	N7—C43—C56—C51	171.5 (3)
N2—C2—C3—C4	−0.7 (5)	C44—C43—C56—C51	−10.7 (5)
C1—C2—C3—C4	171.9 (3)	N9—C57—C58—N10	16.8 (4)
N2—C2—C3—C8	−174.4 (3)	C70—C57—C58—N10	−160.8 (3)
C1—C2—C3—C8	−1.8 (4)	N9—C57—C58—C59	−161.1 (3)
C8—C3—C4—C5	3.3 (5)	C70—C57—C58—C59	21.3 (4)
C2—C3—C4—C5	−170.3 (3)	N10—C58—C59—C60	−17.7 (6)
C3—C4—C5—C6	−0.6 (5)	C57—C58—C59—C60	159.9 (3)
C4—C5—C6—C7	−1.8 (6)	N10—C58—C59—C64	166.4 (3)
C5—C6—C7—C8	1.4 (6)	C57—C58—C59—C64	−16.0 (5)
C6—C7—C8—C3	1.3 (5)	C64—C59—C60—C61	0.6 (6)
C6—C7—C8—C9	−179.2 (3)	C58—C59—C60—C61	−175.3 (4)
C4—C3—C8—C7	−3.6 (5)	C59—C60—C61—C62	−2.1 (6)
C2—C3—C8—C7	170.4 (3)	C60—C61—C62—C63	1.9 (7)
C4—C3—C8—C9	176.9 (3)	C61—C62—C63—C64	−0.2 (7)
C2—C3—C8—C9	−9.1 (4)	C62—C63—C64—C59	−1.3 (6)
C7—C8—C9—C10	13.2 (5)	C62—C63—C64—C65	176.6 (4)
C3—C8—C9—C10	−167.3 (3)	C60—C59—C64—C63	1.1 (5)
C7—C8—C9—C14	−169.4 (3)	C58—C59—C64—C63	177.2 (3)
C3—C8—C9—C14	10.1 (5)	C60—C59—C64—C65	−176.9 (3)
C14—C9—C10—C11	1.0 (5)	C58—C59—C64—C65	−0.8 (5)
C8—C9—C10—C11	178.6 (3)	C63—C64—C65—C66	13.5 (5)
C9—C10—C11—C12	−1.6 (6)	C59—C64—C65—C66	−168.6 (3)
C10—C11—C12—C13	0.8 (5)	C63—C64—C65—C70	−164.5 (3)
C11—C12—C13—C14	0.6 (5)	C59—C64—C65—C70	13.4 (5)
C12—C13—C14—C9	−1.2 (5)	C70—C65—C66—C67	3.0 (6)
C12—C13—C14—C1	−178.7 (3)	C64—C65—C66—C67	−175.1 (4)
C10—C9—C14—C13	0.4 (5)	C65—C66—C67—C68	−0.1 (6)
C8—C9—C14—C13	−177.2 (3)	C66—C67—C68—C69	−2.1 (6)
C10—C9—C14—C1	178.0 (3)	C67—C68—C69—C70	1.3 (6)
C8—C9—C14—C1	0.5 (5)	C66—C65—C70—C69	−3.7 (5)
N1—C1—C14—C13	−14.7 (5)	C64—C65—C70—C69	174.3 (3)
C2—C1—C14—C13	166.2 (3)	C66—C65—C70—C57	174.0 (3)
N1—C1—C14—C9	167.7 (3)	C64—C65—C70—C57	−7.9 (5)
C2—C1—C14—C9	−11.4 (5)	C68—C69—C70—C65	1.6 (5)
N3—C15—C16—N4	26.0 (5)	C68—C69—C70—C57	−176.0 (3)
C28—C15—C16—N4	−154.2 (3)	N9—C57—C70—C65	173.9 (3)
N3—C15—C16—C17	−153.7 (3)	C58—C57—C70—C65	−9.0 (5)
C28—C15—C16—C17	26.0 (4)	N9—C57—C70—C69	−8.5 (6)
N4—C16—C17—C22	166.5 (3)	C58—C57—C70—C69	168.6 (3)
C15—C16—C17—C22	−13.8 (4)	C77—C72—C73—C74	1.6 (7)
N4—C16—C17—C18	−14.7 (4)	C71—C72—C73—C74	−178.3 (5)
C15—C16—C17—C18	165.1 (3)	C72—C73—C74—C75	0.3 (7)
C22—C17—C18—C19	−1.0 (5)	C73—C74—C75—C76	−1.4 (7)
C16—C17—C18—C19	−179.9 (3)	C74—C75—C76—C77	0.5 (8)
C17—C18—C19—C20	1.6 (5)	C75—C76—C77—C72	1.5 (9)
C18—C19—C20—C21	−0.4 (5)	C73—C72—C77—C76	−2.5 (8)
C19—C20—C21—C22	−1.3 (6)	C71—C72—C77—C76	177.3 (6)
C18—C17—C22—C21	−0.6 (5)	C84—C79—C80—C81	0.2 (4)
C16—C17—C22—C21	178.2 (3)	C78—C79—C80—C81	−179.8 (3)
C18—C17—C22—C23	176.3 (3)	C79—C80—C81—C82	−0.6 (6)
C16—C17—C22—C23	−4.9 (5)	C80—C81—C82—C83	0.7 (7)
C20—C21—C22—C17	1.8 (5)	C81—C82—C83—C84	−0.4 (8)
C20—C21—C22—C23	−175.1 (3)	C82—C83—C84—C79	0.1 (7)
C17—C22—C23—C24	−166.6 (3)	C80—C79—C84—C83	0.0 (5)
C21—C22—C23—C24	10.2 (5)	C78—C79—C84—C83	−180.0 (3)
C17—C22—C23—C28	11.0 (5)	C84A—C79A—C80A—C81A	0.3 (6)
C21—C22—C23—C28	−172.2 (3)	C78A—C79A—C80A—C81A	−179.9 (2)
C28—C23—C24—C25	−1.3 (5)	C82A—C81A—C80A—C79A	0.0 (6)
C22—C23—C24—C25	176.4 (3)	C80A—C81A—C82A—C83A	−0.5 (7)
C23—C24—C25—C26	−2.0 (6)	C81A—C82A—C83A—C84A	0.5 (8)
C24—C25—C26—C27	2.8 (6)	C80A—C79A—C84A—C83A	−0.2 (7)
C25—C26—C27—C28	−0.1 (6)	C78A—C79A—C84A—C83A	180.0 (4)
C26—C27—C28—C23	−3.3 (5)	C82A—C83A—C84A—C79A	−0.2 (8)
C26—C27—C28—C15	−178.9 (3)	C14—C1—N1—O1	−1.9 (5)
C24—C23—C28—C27	3.9 (5)	C2—C1—N1—O1	177.3 (3)
C22—C23—C28—C27	−173.8 (3)	C14—C1—N1—Ni1	176.6 (3)
C24—C23—C28—C15	179.6 (3)	C2—C1—N1—Ni1	−4.2 (4)
C22—C23—C28—C15	1.9 (5)	C3—C2—N2—O2	−9.3 (5)
N3—C15—C28—C27	−24.8 (5)	C1—C2—N2—O2	177.6 (3)
C16—C15—C28—C27	155.5 (3)	C3—C2—N2—Ni1	166.7 (3)
N3—C15—C28—C23	159.6 (3)	C1—C2—N2—Ni1	−6.4 (4)
C16—C15—C28—C23	−20.1 (4)	C16—C15—N3—O3	173.6 (3)
N5—C29—C30—N6	28.4 (5)	C28—C15—N3—O3	−6.2 (5)
C42—C29—C30—N6	−150.6 (3)	C16—C15—N3—Ni2	−13.4 (4)
N5—C29—C30—C31	−151.3 (3)	C28—C15—N3—Ni2	166.8 (2)
C42—C29—C30—C31	29.7 (4)	C15—C16—N4—O4	−4.3 (5)
N6—C30—C31—C32	−24.0 (5)	C17—C16—N4—O4	175.4 (3)
C29—C30—C31—C32	155.8 (3)	C30—C29—N5—O5	172.0 (3)
N6—C30—C31—C36	155.9 (3)	C42—C29—N5—O5	−9.0 (5)
C29—C30—C31—C36	−24.4 (5)	C30—C29—N5—Ni1	−18.5 (4)
C36—C31—C32—C33	−1.1 (6)	C42—C29—N5—Ni1	160.5 (3)
C30—C31—C32—C33	178.8 (4)	C29—C30—N6—O6	−4.0 (5)
C31—C32—C33—C34	−0.1 (7)	C31—C30—N6—O6	175.7 (3)
C32—C33—C34—C35	0.8 (7)	C44—C43—N7—O7	178.7 (3)
C33—C34—C35—C36	−0.3 (7)	C56—C43—N7—O7	−3.4 (5)
C32—C31—C36—C35	1.5 (5)	C44—C43—N7—Ni2	−6.0 (4)
C30—C31—C36—C35	−178.3 (3)	C56—C43—N7—Ni2	172.0 (3)
C32—C31—C36—C37	−177.9 (3)	C43—C44—N8—O8	175.6 (3)
C30—C31—C36—C37	2.3 (5)	C45—C44—N8—O8	−8.5 (5)
C34—C35—C36—C31	−0.8 (6)	C43—C44—N8—Ni2	−7.2 (4)
C34—C35—C36—C37	178.5 (4)	C45—C44—N8—Ni2	168.7 (3)
C31—C36—C37—C38	−166.6 (3)	C70—C57—N9—O9	−0.4 (5)
C35—C36—C37—C38	14.1 (5)	C58—C57—N9—O9	−177.7 (3)
C31—C36—C37—C42	14.3 (5)	C70—C57—N9—Ni3	166.5 (3)
C35—C36—C37—C42	−165.1 (3)	C58—C57—N9—Ni3	−10.8 (4)
C42—C37—C38—C39	1.1 (5)	C59—C58—N10—O10	−3.5 (5)
C36—C37—C38—C39	−178.0 (3)	C57—C58—N10—O10	178.9 (3)
C37—C38—C39—C40	0.4 (6)	C59—C58—N10—Ni3	162.1 (3)
C38—C39—C40—C41	−1.6 (6)	C57—C58—N10—Ni3	−15.6 (4)
C39—C40—C41—C42	1.2 (6)	C2—N2—O2—Ni3	143.0 (3)
C40—C41—C42—C37	0.3 (5)	Ni1—N2—O2—Ni3	−32.7 (3)
C40—C41—C42—C29	−173.5 (3)	C16—N4—O4—Ni2	−30.5 (4)
C38—C37—C42—C41	−1.5 (5)	C16—N4—O4—Ni3	148.7 (2)
C36—C37—C42—C41	177.7 (3)	C30—N6—O6—Ni1	−30.9 (4)
C38—C37—C42—C29	172.6 (3)	C30—N6—O6—Ni3	145.4 (2)
C36—C37—C42—C29	−8.3 (5)	C44—N8—O8—Ni3	145.0 (3)
N5—C29—C42—C41	−18.5 (5)	Ni2—N8—O8—Ni3	−32.1 (3)
C30—C29—C42—C41	160.4 (3)	N6—O6—Ni1—N1	−158.8 (6)
N5—C29—C42—C37	167.6 (3)	Ni3—O6—Ni1—N1	24.9 (7)
C30—C29—C42—C37	−13.4 (4)	N6—O6—Ni1—N2	−151.0 (3)
N7—C43—C44—N8	8.4 (4)	Ni3—O6—Ni1—N2	32.64 (14)
C56—C43—C44—N8	−169.7 (3)	N6—O6—Ni1—N5	31.9 (3)
N7—C43—C44—C45	−167.8 (3)	Ni3—O6—Ni1—N5	−144.41 (14)
C56—C43—C44—C45	14.1 (5)	C1—N1—Ni1—O6	8.5 (8)
N8—C44—C45—C46	−6.1 (6)	O1—N1—Ni1—O6	−173.0 (6)
C43—C44—C45—C46	169.4 (3)	C1—N1—Ni1—N2	0.7 (3)
N8—C44—C45—C50	178.3 (3)	O1—N1—Ni1—N2	179.1 (3)
C43—C44—C45—C50	−6.2 (5)	C1—N1—Ni1—N5	177.7 (3)
C50—C45—C46—C47	1.8 (6)	O1—N1—Ni1—N5	−3.9 (3)
C44—C45—C46—C47	−173.7 (4)	C2—N2—Ni1—O6	−175.1 (2)
C45—C46—C47—C48	−1.8 (6)	O2—N2—Ni1—O6	0.8 (2)
C46—C47—C48—C49	1.1 (7)	C2—N2—Ni1—N1	3.5 (2)
C47—C48—C49—C50	−0.4 (7)	O2—N2—Ni1—N1	179.4 (3)
C48—C49—C50—C45	0.4 (6)	C29—N5—Ni1—O6	−5.9 (3)
C48—C49—C50—C51	179.7 (4)	O5—N5—Ni1—O6	163.6 (3)
C46—C45—C50—C49	−1.0 (5)	C29—N5—Ni1—N1	176.0 (3)
C44—C45—C50—C49	174.6 (3)	O5—N5—Ni1—N1	−14.5 (3)
C46—C45—C50—C51	179.7 (3)	N4—O4—Ni2—N8	−149.4 (3)
C44—C45—C50—C51	−4.7 (5)	Ni3—O4—Ni2—N8	31.36 (14)
C49—C50—C51—C56	−171.2 (4)	N4—O4—Ni2—N3	33.3 (3)
C45—C50—C51—C56	8.0 (5)	Ni3—O4—Ni2—N3	−145.90 (14)
C49—C50—C51—C52	9.9 (6)	C43—N7—Ni2—N8	1.8 (3)
C45—C50—C51—C52	−170.8 (3)	O7—N7—Ni2—N8	177.1 (3)
C56—C51—C52—C53	−0.1 (6)	C43—N7—Ni2—N3	179.0 (3)
C50—C51—C52—C53	178.7 (4)	O7—N7—Ni2—N3	−5.7 (3)
C51—C52—C53—C54	0.2 (6)	C44—N8—Ni2—O4	−176.1 (3)
C52—C53—C54—C55	−0.1 (6)	O8—N8—Ni2—O4	1.1 (2)
C53—C54—C55—C56	−0.1 (6)	C44—N8—Ni2—N7	3.3 (2)
C54—C55—C56—C51	0.2 (5)	O8—N8—Ni2—N7	−179.5 (3)
C54—C55—C56—C43	−178.5 (3)	C15—N3—Ni2—O4	−10.2 (3)
C52—C51—C56—C55	−0.1 (5)	O3—N3—Ni2—O4	162.8 (2)
C50—C51—C56—C55	−179.0 (3)	C15—N3—Ni2—N7	170.8 (3)
C52—C51—C56—C43	178.7 (3)	O3—N3—Ni2—N7	−16.1 (2)

### (1) Bis[µ2-9,10-bis(oxidoimino)phenanthrene]bis[µ2-10-(oxidoimino)phenanthrene-9-one oxime](phenanthrene-9,10-dione dioxime)trinickel(II) toluene disolvate Hydrogen-bond geometry (Å, º)

**Table:**

*D*—H···*A*	*D*—H	H···*A*	*D*···*A*	*D*—H···*A*
O1—H1*A*···O5	1.29 (6)	1.13 (6)	2.405 (4)	169 (6)
O3—H2*A*···O7	1.17 (5)	1.23 (5)	2.402 (4)	176 (5)
O9—H3*A*···N4	0.86 (6)	1.82 (6)	2.672 (4)	175 (7)
O10—H4*A*···N6	1.04 (5)	1.63 (5)	2.657 (4)	168 (4)

### (2) Bis(µ2-bis{[10-(oxidoimino)-9,10-dihydrophenanthren-9-ylidene]amino}difluoroborato)(phenanthrene-9,10-dione dioxime)trinickel(II) dichloromethane trisolvate Crystal data

**Table:**

[Ni_3_(C_28_H_16_BF_2_N_4_O_4_)_2_(C_14_H_10_N_2_O_2_)]·3CH_2_Cl_2_	*F*(000) = 2968
*M**_r_* = 1456.88	*D*_x_ = 1.473 Mg m^−^^3^
Monoclinic, *P*2_1_/*c*	Cu *K*α radiation, λ = 1.54184 Å
*a* = 15.6414 (8) Å	Cell parameters from 25728 reflections
*b* = 30.8358 (11) Å	θ = 3.4–76.5°
*c* = 14.7380 (8) Å	µ = 1.67 mm^−^^1^
β = 112.411 (6)°	*T* = 100 K
*V* = 6571.5 (6) Å^3^	Block, red
*Z* = 4	0.29 × 0.07 × 0.04 mm

### (2) Bis(µ2-bis{[10-(oxidoimino)-9,10-dihydrophenanthren-9-ylidene]amino}difluoroborato)(phenanthrene-9,10-dione dioxime)trinickel(II) dichloromethane trisolvate Data collection

**Table:**

Agilent SuperNova Dual Source diffractometer with an Atlas detector	13041 independent reflections
Radiation source: sealed X-ray tube	8782 reflections with *I* > 2σ(*I*)
Detector resolution: 5.2940 pixels mm^-1^	*R*_int_ = 0.060
ω scans	θ_max_ = 75.0°, θ_min_ = 3.4°
Absorption correction: multi-scan (CrysAlis PRO; Agilent, 2014)	*h* = −19→19
*T*_min_ = 0.303, *T*_max_ = 1.000	*k* = −26→38
25337 measured reflections	*l* = −18→18

### (2) Bis(µ2-bis{[10-(oxidoimino)-9,10-dihydrophenanthren-9-ylidene]amino}difluoroborato)(phenanthrene-9,10-dione dioxime)trinickel(II) dichloromethane trisolvate Refinement

**Table:**

Refinement on *F*^2^	0 restraints
Least-squares matrix: full	Hydrogen site location: inferred from neighbouring sites
*R*[*F*^2^ > 2σ(*F*^2^)] = 0.077	H atoms treated by a mixture of independent and constrained refinement
*wR*(*F*^2^) = 0.226	*w* = 1/[σ^2^(*F*_o_^2^) + (0.108*P*)^2^ + 8.2009*P*] where *P* = (*F*_o_^2^ + 2*F*_c_^2^)/3
*S* = 1.03	(Δ/σ)_max_ = 0.006
13041 reflections	Δρ_max_ = 1.16 e Å^−^^3^
896 parameters	Δρ_min_ = −0.83 e Å^−^^3^

### (2) Bis(µ2-bis{[10-(oxidoimino)-9,10-dihydrophenanthren-9-ylidene]amino}difluoroborato)(phenanthrene-9,10-dione dioxime)trinickel(II) dichloromethane trisolvate Special details

**Table:**

Experimental. CrysAlisPro. (Agilent, 2014). Empirical absorption correction using spherical harmonics, implemented in SCALE3 ABSPACK scaling algorithm.
Geometry. All esds (except the esd in the dihedral angle between two l.s. planes) are estimated using the full covariance matrix. The cell esds are taken into account individually in the estimation of esds in distances, angles and torsion angles; correlations between esds in cell parameters are only used when they are defined by crystal symmetry. An approximate (isotropic) treatment of cell esds is used for estimating esds involving l.s. planes.
Refinement. Refinement of F^2^ against ALL reflections. The weighted R-factor wR and goodness of fit S are based on F^2^, conventional R-factors R are based on F, with F set to zero for negative F^2^. The threshold expression of F^2^ > 2sigma(F^2^) is used only for calculating R-factors(gt) etc. and is not relevant to the choice of reflections for refinement. R-factors based on F^2^ are statistically about twice as large as those based on F, and R- factors based on ALL data will be even larger. Three molecules of what appeared to be dichloromethane were found to be badly disordered. Attempts to model the disorder were unsatisfactory. The contributions to the scattering factors due to these solvent molecules were removed by use of the utility SQUEEZE (Sluis and Spek, 1990) in PLATON98 (Spek, 1998). PLATON98 was used as incorporated in WinGX (Farrugia, 1999).

### (2) Bis(µ2-bis{[10-(oxidoimino)-9,10-dihydrophenanthren-9-ylidene]amino}difluoroborato)(phenanthrene-9,10-dione dioxime)trinickel(II) dichloromethane trisolvate Fractional atomic coordinates and isotropic or equivalent isotropic displacement parameters (Å^2^)

**Table:**

	*x*	*y*	*z*	*U*_iso_*/*U*_eq_	
C1	0.3411 (3)	0.33690 (15)	0.7321 (4)	0.0323 (10)	
C2	0.4326 (3)	0.33998 (16)	0.7254 (4)	0.0336 (11)	
C3	0.4765 (4)	0.29952 (16)	0.7127 (4)	0.0347 (11)	
C4	0.5720 (4)	0.29722 (18)	0.7393 (4)	0.0423 (12)	
H4	0.6093	0.3220	0.7661	0.051*	
C5	0.6130 (4)	0.25876 (18)	0.7267 (5)	0.0499 (14)	
H5	0.6781	0.2571	0.7446	0.060*	
C6	0.5574 (4)	0.22266 (18)	0.6875 (5)	0.0498 (15)	
H6	0.5847	0.1963	0.6785	0.060*	
C7	0.4637 (4)	0.22483 (17)	0.6619 (4)	0.0460 (14)	
H7	0.4274	0.1997	0.6361	0.055*	
C8	0.4198 (4)	0.26291 (15)	0.6727 (4)	0.0362 (11)	
C9	0.3201 (4)	0.26641 (15)	0.6448 (4)	0.0357 (11)	
C10	0.2594 (4)	0.23476 (16)	0.5841 (4)	0.0394 (12)	
H10	0.2844	0.2106	0.5627	0.047*	
C11	0.1655 (4)	0.23823 (18)	0.5555 (4)	0.0438 (13)	
H11	0.1267	0.2162	0.5158	0.053*	
C12	0.1264 (4)	0.27305 (18)	0.5831 (4)	0.0422 (12)	
H12	0.0611	0.2754	0.5620	0.051*	
C13	0.1837 (4)	0.30485 (16)	0.6426 (4)	0.0361 (11)	
H13	0.1571	0.3291	0.6619	0.043*	
C14	0.2797 (4)	0.30177 (15)	0.6744 (4)	0.0336 (11)	
C15	0.2126 (3)	0.36498 (16)	0.4672 (3)	0.0310 (10)	
C16	0.3085 (3)	0.37760 (15)	0.4870 (3)	0.0295 (10)	
C17	0.3700 (3)	0.34790 (16)	0.4634 (3)	0.0304 (10)	
C18	0.4630 (3)	0.35767 (17)	0.4827 (4)	0.0352 (11)	
H18	0.4869	0.3853	0.5083	0.042*	
C19	0.5208 (4)	0.32743 (18)	0.4649 (4)	0.0442 (13)	
H19	0.5832	0.3346	0.4770	0.053*	
C20	0.4864 (4)	0.28685 (19)	0.4294 (4)	0.0472 (14)	
H20	0.5261	0.2657	0.4196	0.057*	
C21	0.3950 (4)	0.27698 (18)	0.4081 (4)	0.0423 (12)	
H21	0.3725	0.2491	0.3827	0.051*	
C22	0.3341 (3)	0.30700 (16)	0.4230 (4)	0.0330 (10)	
C23	0.2352 (4)	0.29719 (16)	0.3911 (4)	0.0335 (10)	
C24	0.1973 (4)	0.25910 (18)	0.3381 (4)	0.0423 (12)	
H24	0.2378	0.2386	0.3275	0.051*	
C25	0.1044 (4)	0.25044 (19)	0.3013 (4)	0.0464 (13)	
H25	0.0816	0.2242	0.2667	0.056*	
C26	0.0436 (4)	0.28018 (19)	0.3149 (4)	0.0455 (13)	
H26	−0.0211	0.2750	0.2874	0.055*	
C27	0.0781 (4)	0.31762 (18)	0.3690 (4)	0.0380 (11)	
H27	0.0365	0.3376	0.3794	0.046*	
C28	0.1730 (3)	0.32636 (16)	0.4084 (4)	0.0341 (11)	
C29	0.3296 (3)	0.47670 (15)	0.9399 (4)	0.0325 (10)	
C30	0.3915 (3)	0.50074 (15)	0.9040 (3)	0.0316 (10)	
C31	0.4137 (3)	0.54622 (15)	0.9300 (3)	0.0322 (10)	
C32	0.4728 (4)	0.57048 (16)	0.8987 (4)	0.0386 (12)	
H32	0.4988	0.5573	0.8569	0.046*	
C33	0.4942 (4)	0.61279 (17)	0.9267 (4)	0.0448 (13)	
H33	0.5354	0.6284	0.9053	0.054*	
C34	0.4558 (5)	0.63252 (18)	0.9862 (5)	0.0557 (17)	
H34	0.4704	0.6618	1.0061	0.067*	
C35	0.3969 (4)	0.60974 (17)	1.0163 (4)	0.0454 (14)	
H35	0.3712	0.6239	1.0573	0.055*	
C36	0.3721 (4)	0.56675 (16)	0.9903 (3)	0.0340 (11)	
C37	0.3088 (4)	0.54261 (16)	1.0242 (4)	0.0360 (11)	
C38	0.2666 (4)	0.56390 (18)	1.0798 (4)	0.0418 (13)	
H38	0.2772	0.5941	1.0922	0.050*	
C39	0.2105 (4)	0.5425 (2)	1.1168 (4)	0.0497 (15)	
H39	0.1828	0.5579	1.1545	0.060*	
C40	0.1938 (4)	0.4987 (2)	1.0998 (4)	0.0443 (13)	
H40	0.1552	0.4838	1.1261	0.053*	
C41	0.2339 (4)	0.47662 (17)	1.0439 (4)	0.0372 (11)	
H41	0.2230	0.4464	1.0326	0.045*	
C42	0.2897 (4)	0.49811 (16)	1.0043 (3)	0.0355 (11)	
C43	0.1661 (3)	0.49600 (19)	0.6807 (4)	0.0400 (12)	
C44	0.2317 (3)	0.52960 (18)	0.6755 (4)	0.0392 (12)	
C45	0.2153 (4)	0.5756 (2)	0.6941 (4)	0.0511 (16)	
C46	0.2581 (4)	0.6091 (2)	0.6633 (5)	0.0581 (18)	
H46	0.2979	0.6023	0.6301	0.070*	
C47	0.2434 (5)	0.6522 (2)	0.6804 (7)	0.077 (3)	
H47	0.2730	0.6749	0.6597	0.093*	
C48	0.1844 (5)	0.6615 (3)	0.7286 (7)	0.094 (3)	
H48	0.1729	0.6908	0.7403	0.112*	
C49	0.1427 (5)	0.6285 (3)	0.7595 (6)	0.083 (3)	
H49	0.1032	0.6356	0.7928	0.100*	
C50	0.1571 (4)	0.5845 (2)	0.7429 (5)	0.061 (2)	
C51	0.1151 (4)	0.5488 (3)	0.7768 (4)	0.062 (2)	
C52	0.0734 (5)	0.5578 (3)	0.8439 (5)	0.073 (2)	
H52	0.0721	0.5866	0.8658	0.088*	
C53	0.0348 (5)	0.5250 (4)	0.8776 (5)	0.088 (3)	
H53	0.0047	0.5317	0.9209	0.105*	
C54	0.0385 (4)	0.4820 (4)	0.8502 (5)	0.077 (3)	
H54	0.0127	0.4596	0.8761	0.093*	
C55	0.0804 (4)	0.4722 (3)	0.7841 (4)	0.0604 (19)	
H55	0.0838	0.4430	0.7650	0.072*	
C56	0.1175 (4)	0.5059 (2)	0.7462 (4)	0.0500 (16)	
C57	0.6326 (3)	0.48466 (16)	0.6652 (4)	0.0315 (10)	
C58	0.5935 (3)	0.52870 (16)	0.6698 (3)	0.0315 (10)	
C59	0.6591 (3)	0.56487 (15)	0.7051 (3)	0.0304 (10)	
C60	0.6390 (4)	0.60134 (17)	0.7479 (4)	0.0395 (12)	
H60	0.5788	0.6050	0.7481	0.047*	
C61	0.7056 (4)	0.63235 (18)	0.7902 (5)	0.0463 (13)	
H61	0.6915	0.6570	0.8207	0.056*	
C62	0.7926 (4)	0.62788 (17)	0.7885 (4)	0.0436 (13)	
H62	0.8380	0.6496	0.8171	0.052*	
C63	0.8141 (4)	0.59185 (17)	0.7452 (4)	0.0363 (11)	
H63	0.8743	0.5889	0.7446	0.044*	
C64	0.7473 (3)	0.55957 (15)	0.7021 (3)	0.0298 (10)	
C65	0.7705 (3)	0.52067 (16)	0.6569 (3)	0.0320 (10)	
C66	0.8480 (3)	0.51969 (17)	0.6315 (4)	0.0346 (11)	
H66	0.8867	0.5445	0.6430	0.041*	
C67	0.8690 (3)	0.48300 (19)	0.5900 (4)	0.0390 (12)	
H67	0.9216	0.4828	0.5728	0.047*	
C68	0.8134 (4)	0.44667 (18)	0.5735 (4)	0.0409 (12)	
H68	0.8281	0.4215	0.5450	0.049*	
C69	0.7366 (3)	0.44671 (18)	0.5982 (4)	0.0389 (12)	
H69	0.6988	0.4216	0.5867	0.047*	
C70	0.7146 (3)	0.48316 (16)	0.6398 (4)	0.0332 (11)	
N1	0.3219 (3)	0.36493 (12)	0.7882 (3)	0.0321 (9)	
N2	0.4789 (3)	0.37504 (13)	0.7289 (3)	0.0339 (9)	
N3	0.1712 (3)	0.39220 (13)	0.5053 (3)	0.0316 (9)	
N4	0.3296 (3)	0.41530 (13)	0.5315 (3)	0.0290 (8)	
N5	0.3158 (3)	0.43671 (13)	0.9063 (3)	0.0337 (9)	
N6	0.4215 (3)	0.47684 (11)	0.8493 (3)	0.0288 (8)	
N7	0.1558 (3)	0.46191 (15)	0.6258 (3)	0.0364 (10)	
N8	0.3047 (3)	0.52333 (14)	0.6565 (3)	0.0359 (9)	
N9	0.5880 (3)	0.45401 (13)	0.6849 (3)	0.0346 (9)	
N10	0.5047 (3)	0.52610 (13)	0.6498 (3)	0.0378 (10)	
O1	0.2406 (3)	0.35856 (11)	0.8007 (3)	0.0397 (8)	
O2	0.4437 (2)	0.41270 (10)	0.7481 (3)	0.0329 (7)	
O3	0.0833 (2)	0.38339 (12)	0.4931 (3)	0.0436 (9)	
O4	0.4102 (2)	0.43359 (11)	0.5495 (2)	0.0319 (7)	
O5	0.2580 (3)	0.41112 (11)	0.9310 (3)	0.0422 (9)	
O6	0.4795 (2)	0.49203 (10)	0.8119 (3)	0.0324 (7)	
O7	0.0898 (3)	0.43277 (13)	0.6260 (3)	0.0448 (9)	
O8	0.3220 (2)	0.48291 (11)	0.6323 (3)	0.0347 (8)	
O9	0.6191 (2)	0.41229 (11)	0.6839 (3)	0.0373 (8)	
H1A	0.582 (4)	0.3945 (14)	0.694 (5)	0.056*	
O10	0.4608 (3)	0.56409 (12)	0.6526 (3)	0.0463 (9)	
H2A	0.395 (5)	0.5572 (5)	0.654 (5)	0.069*	
Ni1	0.37718 (6)	0.41976 (2)	0.82564 (6)	0.0306 (2)	
Ni2	0.23963 (5)	0.44031 (3)	0.57163 (6)	0.0301 (2)	
Ni3	0.45817 (5)	0.46682 (2)	0.67739 (6)	0.0277 (2)	
F1	0.3252 (3)	0.34124 (11)	0.9649 (3)	0.0556 (9)	
F2	0.1678 (3)	0.35125 (12)	0.9084 (3)	0.0601 (10)	
F3	−0.0369 (2)	0.39118 (12)	0.5394 (3)	0.0613 (10)	
F4	0.0009 (2)	0.44772 (11)	0.4612 (3)	0.0506 (8)	
B1	0.2493 (5)	0.3650 (2)	0.9034 (6)	0.0486 (16)	
B2	0.0335 (4)	0.4143 (2)	0.5275 (6)	0.0437 (15)	

### (2) Bis(µ2-bis{[10-(oxidoimino)-9,10-dihydrophenanthren-9-ylidene]amino}difluoroborato)(phenanthrene-9,10-dione dioxime)trinickel(II) dichloromethane trisolvate Atomic displacement parameters (Å^2^)

**Table:**

	*U*^11^	*U*^22^	*U*^33^	*U*^12^	*U*^13^	*U*^23^
C1	0.044 (3)	0.021 (2)	0.029 (2)	0.0022 (19)	0.011 (2)	0.0065 (19)
C2	0.042 (3)	0.026 (2)	0.030 (3)	0.002 (2)	0.011 (2)	0.004 (2)
C3	0.052 (3)	0.023 (2)	0.028 (2)	0.003 (2)	0.014 (2)	0.000 (2)
C4	0.051 (3)	0.030 (3)	0.042 (3)	0.005 (2)	0.013 (2)	−0.002 (2)
C5	0.058 (3)	0.035 (3)	0.052 (4)	0.010 (3)	0.015 (3)	−0.004 (3)
C6	0.064 (4)	0.029 (3)	0.055 (4)	0.017 (3)	0.022 (3)	−0.001 (3)
C7	0.068 (4)	0.024 (3)	0.038 (3)	0.001 (2)	0.012 (3)	−0.002 (2)
C8	0.061 (3)	0.016 (2)	0.029 (3)	0.001 (2)	0.015 (2)	0.0017 (19)
C9	0.058 (3)	0.020 (2)	0.028 (2)	−0.002 (2)	0.014 (2)	0.006 (2)
C10	0.065 (3)	0.019 (2)	0.032 (3)	−0.006 (2)	0.016 (2)	0.002 (2)
C11	0.059 (3)	0.033 (3)	0.035 (3)	−0.016 (2)	0.014 (2)	0.001 (2)
C12	0.054 (3)	0.036 (3)	0.036 (3)	−0.010 (2)	0.015 (2)	0.002 (2)
C13	0.049 (3)	0.028 (3)	0.031 (3)	−0.005 (2)	0.015 (2)	0.003 (2)
C14	0.049 (3)	0.023 (2)	0.024 (2)	−0.006 (2)	0.010 (2)	0.0060 (19)
C15	0.036 (2)	0.032 (3)	0.023 (2)	0.0051 (19)	0.0079 (18)	0.006 (2)
C16	0.041 (2)	0.026 (2)	0.025 (2)	0.0059 (19)	0.0159 (19)	0.0028 (19)
C17	0.041 (2)	0.029 (2)	0.022 (2)	0.0046 (19)	0.0133 (19)	0.0010 (19)
C18	0.039 (2)	0.034 (3)	0.032 (3)	0.001 (2)	0.013 (2)	−0.002 (2)
C19	0.041 (3)	0.041 (3)	0.049 (3)	0.006 (2)	0.016 (2)	−0.007 (3)
C20	0.050 (3)	0.041 (3)	0.050 (3)	0.011 (2)	0.018 (3)	−0.010 (3)
C21	0.048 (3)	0.033 (3)	0.044 (3)	0.001 (2)	0.015 (2)	−0.010 (2)
C22	0.045 (3)	0.030 (3)	0.025 (2)	0.002 (2)	0.015 (2)	−0.002 (2)
C23	0.048 (3)	0.029 (3)	0.026 (2)	0.000 (2)	0.016 (2)	0.003 (2)
C24	0.054 (3)	0.038 (3)	0.040 (3)	−0.005 (2)	0.023 (3)	−0.009 (2)
C25	0.061 (3)	0.042 (3)	0.038 (3)	−0.013 (3)	0.021 (3)	−0.014 (3)
C26	0.050 (3)	0.049 (3)	0.033 (3)	−0.011 (3)	0.012 (2)	−0.005 (3)
C27	0.043 (3)	0.037 (3)	0.033 (3)	0.002 (2)	0.013 (2)	0.004 (2)
C28	0.042 (3)	0.029 (2)	0.028 (2)	0.002 (2)	0.010 (2)	0.008 (2)
C29	0.045 (3)	0.023 (2)	0.026 (2)	0.0069 (19)	0.011 (2)	0.0031 (19)
C30	0.042 (2)	0.024 (2)	0.025 (2)	0.0043 (19)	0.0094 (19)	0.0014 (19)
C31	0.048 (3)	0.021 (2)	0.021 (2)	0.0047 (19)	0.0066 (19)	0.0026 (19)
C32	0.058 (3)	0.025 (2)	0.030 (3)	0.006 (2)	0.014 (2)	−0.001 (2)
C33	0.072 (4)	0.023 (3)	0.039 (3)	−0.004 (2)	0.021 (3)	−0.001 (2)
C34	0.095 (5)	0.020 (3)	0.051 (4)	−0.003 (3)	0.027 (3)	−0.009 (3)
C35	0.075 (4)	0.028 (3)	0.033 (3)	0.006 (3)	0.020 (3)	−0.006 (2)
C36	0.050 (3)	0.024 (2)	0.023 (2)	0.003 (2)	0.009 (2)	−0.0012 (19)
C37	0.045 (3)	0.032 (3)	0.021 (2)	0.009 (2)	0.002 (2)	0.001 (2)
C38	0.053 (3)	0.038 (3)	0.028 (3)	0.009 (2)	0.008 (2)	−0.009 (2)
C39	0.065 (4)	0.054 (4)	0.030 (3)	0.014 (3)	0.018 (3)	−0.008 (3)
C40	0.054 (3)	0.054 (4)	0.023 (2)	0.011 (3)	0.011 (2)	0.004 (2)
C41	0.046 (3)	0.031 (3)	0.033 (3)	0.004 (2)	0.013 (2)	0.004 (2)
C42	0.052 (3)	0.028 (3)	0.019 (2)	0.010 (2)	0.006 (2)	0.0008 (19)
C43	0.032 (2)	0.056 (3)	0.026 (2)	0.014 (2)	0.0044 (19)	−0.007 (2)
C44	0.032 (2)	0.046 (3)	0.031 (3)	0.011 (2)	0.003 (2)	−0.010 (2)
C45	0.036 (3)	0.057 (4)	0.045 (3)	0.017 (3)	−0.002 (2)	−0.021 (3)
C46	0.043 (3)	0.039 (3)	0.074 (5)	0.008 (2)	0.002 (3)	−0.024 (3)
C47	0.052 (4)	0.056 (4)	0.103 (6)	0.009 (3)	0.006 (4)	−0.036 (4)
C48	0.054 (4)	0.070 (5)	0.123 (8)	0.019 (4)	−0.005 (4)	−0.062 (5)
C49	0.053 (4)	0.093 (6)	0.082 (6)	0.024 (4)	0.001 (4)	−0.048 (5)
C50	0.037 (3)	0.080 (5)	0.048 (4)	0.018 (3)	−0.003 (3)	−0.034 (4)
C51	0.043 (3)	0.104 (6)	0.028 (3)	0.023 (3)	0.002 (2)	−0.024 (3)
C52	0.045 (3)	0.129 (7)	0.036 (3)	0.022 (4)	0.005 (3)	−0.028 (4)
C53	0.041 (3)	0.186 (11)	0.031 (3)	0.028 (5)	0.007 (3)	−0.022 (5)
C54	0.045 (3)	0.155 (9)	0.028 (3)	0.007 (4)	0.009 (3)	−0.001 (4)
C55	0.037 (3)	0.115 (6)	0.025 (3)	0.006 (3)	0.007 (2)	−0.010 (3)
C56	0.031 (2)	0.087 (5)	0.027 (3)	0.013 (3)	0.006 (2)	−0.012 (3)
C57	0.034 (2)	0.031 (3)	0.027 (2)	0.0035 (19)	0.0096 (19)	−0.003 (2)
C58	0.037 (2)	0.034 (3)	0.026 (2)	0.000 (2)	0.0141 (19)	−0.002 (2)
C59	0.041 (2)	0.027 (2)	0.025 (2)	0.0019 (19)	0.0137 (19)	0.0026 (19)
C60	0.054 (3)	0.032 (3)	0.037 (3)	−0.002 (2)	0.023 (2)	0.001 (2)
C61	0.065 (4)	0.031 (3)	0.048 (3)	0.000 (3)	0.028 (3)	−0.007 (3)
C62	0.061 (3)	0.027 (3)	0.042 (3)	−0.012 (2)	0.018 (3)	−0.004 (2)
C63	0.044 (3)	0.034 (3)	0.030 (3)	−0.004 (2)	0.012 (2)	0.005 (2)
C64	0.041 (2)	0.028 (2)	0.018 (2)	0.0001 (19)	0.0091 (18)	0.0051 (19)
C65	0.041 (2)	0.031 (3)	0.022 (2)	0.001 (2)	0.0104 (19)	0.0010 (19)
C66	0.037 (2)	0.037 (3)	0.028 (2)	0.000 (2)	0.0108 (19)	0.006 (2)
C67	0.033 (2)	0.052 (3)	0.031 (3)	0.009 (2)	0.011 (2)	0.005 (2)
C68	0.044 (3)	0.042 (3)	0.038 (3)	0.002 (2)	0.017 (2)	−0.011 (2)
C69	0.039 (3)	0.041 (3)	0.036 (3)	−0.004 (2)	0.013 (2)	−0.009 (2)
C70	0.032 (2)	0.034 (3)	0.030 (2)	−0.0023 (19)	0.0083 (19)	−0.003 (2)
N1	0.042 (2)	0.0176 (18)	0.039 (2)	−0.0029 (16)	0.0183 (18)	0.0038 (17)
N2	0.047 (2)	0.020 (2)	0.034 (2)	0.0032 (17)	0.0156 (18)	−0.0009 (17)
N3	0.0329 (19)	0.029 (2)	0.032 (2)	−0.0009 (16)	0.0119 (17)	0.0026 (17)
N4	0.0354 (19)	0.027 (2)	0.026 (2)	0.0023 (16)	0.0130 (16)	0.0018 (16)
N5	0.053 (2)	0.0196 (19)	0.032 (2)	0.0056 (17)	0.0206 (19)	0.0067 (17)
N6	0.038 (2)	0.0146 (17)	0.032 (2)	−0.0032 (15)	0.0109 (16)	0.0013 (16)
N7	0.034 (2)	0.046 (3)	0.029 (2)	0.0027 (18)	0.0125 (17)	0.0015 (19)
N8	0.038 (2)	0.032 (2)	0.033 (2)	0.0069 (17)	0.0086 (17)	−0.0043 (18)
N9	0.042 (2)	0.027 (2)	0.036 (2)	−0.0039 (17)	0.0156 (18)	−0.0092 (18)
N10	0.052 (2)	0.025 (2)	0.040 (2)	0.0038 (18)	0.021 (2)	−0.0010 (19)
O1	0.049 (2)	0.0295 (18)	0.046 (2)	−0.0043 (15)	0.0243 (17)	−0.0047 (16)
O2	0.055 (2)	0.0144 (15)	0.0378 (19)	0.0011 (13)	0.0274 (16)	0.0017 (14)
O3	0.0344 (18)	0.039 (2)	0.058 (2)	−0.0016 (15)	0.0185 (17)	−0.0068 (19)
O4	0.0332 (16)	0.0310 (17)	0.0326 (18)	−0.0019 (13)	0.0137 (14)	−0.0042 (15)
O5	0.064 (2)	0.0268 (18)	0.047 (2)	−0.0021 (16)	0.034 (2)	0.0006 (17)
O6	0.0429 (18)	0.0201 (15)	0.0347 (18)	−0.0032 (13)	0.0155 (15)	−0.0033 (14)
O7	0.043 (2)	0.054 (2)	0.043 (2)	−0.0077 (17)	0.0235 (17)	−0.0037 (19)
O8	0.0345 (16)	0.0273 (17)	0.041 (2)	0.0049 (13)	0.0123 (15)	−0.0044 (15)
O9	0.0384 (18)	0.0263 (17)	0.048 (2)	0.0018 (14)	0.0167 (16)	−0.0074 (16)
O10	0.057 (2)	0.0255 (18)	0.059 (3)	0.0003 (16)	0.025 (2)	0.0035 (18)
Ni1	0.0451 (5)	0.0169 (4)	0.0317 (4)	0.0006 (3)	0.0168 (4)	0.0009 (3)
Ni2	0.0342 (4)	0.0274 (4)	0.0293 (4)	0.0031 (3)	0.0127 (3)	−0.0014 (3)
Ni3	0.0348 (4)	0.0182 (4)	0.0311 (4)	0.0013 (3)	0.0137 (3)	0.0008 (3)
F1	0.071 (2)	0.0393 (18)	0.058 (2)	0.0011 (16)	0.0272 (18)	0.0146 (16)
F2	0.072 (2)	0.048 (2)	0.072 (3)	−0.0111 (17)	0.041 (2)	−0.0011 (19)
F3	0.053 (2)	0.056 (2)	0.083 (3)	−0.0035 (16)	0.0350 (19)	−0.004 (2)
F4	0.0507 (18)	0.0436 (19)	0.049 (2)	0.0051 (15)	0.0097 (15)	−0.0025 (16)
B1	0.061 (4)	0.037 (4)	0.051 (4)	−0.002 (3)	0.025 (3)	−0.001 (3)
B2	0.045 (3)	0.035 (3)	0.059 (4)	−0.005 (3)	0.028 (3)	−0.003 (3)

### (2) Bis(µ2-bis{[10-(oxidoimino)-9,10-dihydrophenanthren-9-ylidene]amino}difluoroborato)(phenanthrene-9,10-dione dioxime)trinickel(II) dichloromethane trisolvate Geometric parameters (Å, º)

**Table:**

C1—N1	1.307 (6)	C44—C45	1.485 (8)
C1—C2	1.475 (7)	C45—C50	1.385 (9)
C1—C14	1.482 (7)	C45—C46	1.399 (10)
C2—N2	1.291 (6)	C46—C47	1.388 (9)
C2—C3	1.470 (7)	C46—H46	0.9500
C3—C4	1.394 (7)	C47—C48	1.392 (12)
C3—C8	1.418 (7)	C47—H47	0.9500
C4—C5	1.394 (7)	C48—C49	1.375 (14)
C4—H4	0.9500	C48—H48	0.9500
C5—C6	1.396 (9)	C49—C50	1.413 (10)
C5—H5	0.9500	C49—H49	0.9500
C6—C7	1.367 (8)	C50—C51	1.464 (11)
C6—H6	0.9500	C51—C52	1.403 (9)
C7—C8	1.400 (7)	C51—C56	1.405 (10)
C7—H7	0.9500	C52—C53	1.364 (13)
C8—C9	1.458 (8)	C52—H52	0.9500
C9—C14	1.410 (7)	C53—C54	1.395 (13)
C9—C10	1.417 (7)	C53—H53	0.9500
C10—C11	1.369 (8)	C54—C55	1.399 (9)
C10—H10	0.9500	C54—H54	0.9500
C11—C12	1.372 (8)	C55—C56	1.404 (10)
C11—H11	0.9500	C55—H55	0.9500
C12—C13	1.391 (7)	C57—N9	1.273 (6)
C12—H12	0.9500	C57—C70	1.466 (7)
C13—C14	1.395 (7)	C57—C58	1.502 (7)
C13—H13	0.9500	C58—N10	1.308 (6)
C15—N3	1.309 (6)	C58—C59	1.470 (7)
C15—C28	1.464 (7)	C59—C60	1.383 (7)
C15—C16	1.467 (7)	C59—C64	1.407 (7)
C16—N4	1.314 (6)	C60—C61	1.376 (8)
C16—C17	1.464 (6)	C60—H60	0.9500
C17—C18	1.404 (7)	C61—C62	1.377 (8)
C17—C22	1.416 (7)	C61—H61	0.9500
C18—C19	1.391 (7)	C62—C63	1.385 (8)
C18—H18	0.9500	C62—H62	0.9500
C19—C20	1.384 (8)	C63—C64	1.407 (7)
C19—H19	0.9500	C63—H63	0.9500
C20—C21	1.376 (8)	C64—C65	1.483 (7)
C20—H20	0.9500	C65—C66	1.398 (7)
C21—C22	1.404 (7)	C65—C70	1.414 (7)
C21—H21	0.9500	C66—C67	1.384 (7)
C22—C23	1.465 (7)	C66—H66	0.9500
C23—C24	1.410 (7)	C67—C68	1.381 (8)
C23—C28	1.418 (7)	C67—H67	0.9500
C24—C25	1.370 (8)	C68—C69	1.382 (7)
C24—H24	0.9500	C68—H68	0.9500
C25—C26	1.389 (8)	C69—C70	1.385 (7)
C25—H25	0.9500	C69—H69	0.9500
C26—C27	1.390 (8)	N1—O1	1.367 (5)
C26—H26	0.9500	N1—Ni1	1.883 (4)
C27—C28	1.399 (7)	N2—O2	1.360 (5)
C27—H27	0.9500	N3—O3	1.346 (5)
C29—N5	1.315 (6)	N3—Ni2	1.872 (4)
C29—C30	1.469 (7)	N4—O4	1.312 (5)
C29—C42	1.476 (7)	N4—Ni2	1.887 (4)
C30—N6	1.304 (6)	N5—O5	1.350 (5)
C30—C31	1.460 (7)	N5—Ni1	1.865 (4)
C31—C32	1.398 (8)	N6—O6	1.314 (5)
C31—C36	1.432 (7)	N6—Ni1	1.875 (4)
C32—C33	1.371 (7)	N7—O7	1.370 (6)
C32—H32	0.9500	N7—Ni2	1.897 (4)
C33—C34	1.379 (8)	N8—O8	1.352 (5)
C33—H33	0.9500	N9—O9	1.378 (5)
C34—C35	1.361 (9)	N9—Ni3	2.029 (4)
C34—H34	0.9500	N10—O10	1.367 (5)
C35—C36	1.393 (7)	N10—Ni3	2.064 (4)
C35—H35	0.9500	O1—B1	1.480 (8)
C36—C37	1.470 (8)	O2—Ni1	1.829 (3)
C37—C38	1.397 (7)	O2—Ni3	2.025 (3)
C37—C42	1.411 (7)	O3—B2	1.440 (7)
C38—C39	1.368 (9)	O4—Ni3	2.022 (3)
C38—H38	0.9500	O5—B1	1.471 (8)
C39—C40	1.379 (9)	O6—Ni3	2.036 (3)
C39—H39	0.9500	O7—B2	1.493 (8)
C40—C41	1.390 (8)	O8—Ni2	1.819 (3)
C40—H40	0.9500	O8—Ni3	2.036 (3)
C41—C42	1.389 (8)	O9—H1A	0.85 (7)
C41—H41	0.9500	O10—H2A	1.06 (7)
C43—N7	1.298 (7)	F1—B1	1.395 (8)
C43—C56	1.471 (7)	F2—B1	1.372 (8)
C43—C44	1.481 (8)	F3—B2	1.378 (7)
C44—N8	1.290 (7)	F4—B2	1.377 (8)
			
N1—C1—C2	117.6 (4)	C48—C49—H49	119.1
N1—C1—C14	126.3 (5)	C50—C49—H49	119.1
C2—C1—C14	116.2 (4)	C45—C50—C49	117.4 (8)
N2—C2—C3	115.7 (5)	C45—C50—C51	119.8 (6)
N2—C2—C1	126.5 (4)	C49—C50—C51	122.8 (7)
C3—C2—C1	117.8 (4)	C52—C51—C56	119.2 (8)
C4—C3—C8	120.7 (5)	C52—C51—C50	118.9 (7)
C4—C3—C2	120.7 (5)	C56—C51—C50	121.9 (6)
C8—C3—C2	118.7 (5)	C53—C52—C51	120.0 (8)
C3—C4—C5	120.3 (5)	C53—C52—H52	120.0
C3—C4—H4	119.9	C51—C52—H52	120.0
C5—C4—H4	119.9	C52—C53—C54	121.7 (7)
C4—C5—C6	119.2 (6)	C52—C53—H53	119.1
C4—C5—H5	120.4	C54—C53—H53	119.1
C6—C5—H5	120.4	C53—C54—C55	119.2 (9)
C7—C6—C5	120.6 (5)	C53—C54—H54	120.4
C7—C6—H6	119.7	C55—C54—H54	120.4
C5—C6—H6	119.7	C54—C55—C56	119.5 (8)
C6—C7—C8	122.0 (5)	C54—C55—H55	120.2
C6—C7—H7	119.0	C56—C55—H55	120.2
C8—C7—H7	119.0	C55—C56—C51	120.2 (6)
C7—C8—C3	117.3 (5)	C55—C56—C43	120.3 (6)
C7—C8—C9	123.3 (5)	C51—C56—C43	119.3 (6)
C3—C8—C9	119.4 (4)	N9—C57—C70	130.1 (5)
C14—C9—C10	117.2 (5)	N9—C57—C58	113.1 (4)
C14—C9—C8	121.8 (4)	C70—C57—C58	116.8 (4)
C10—C9—C8	121.0 (5)	N10—C58—C59	131.6 (4)
C11—C10—C9	121.5 (5)	N10—C58—C57	110.5 (4)
C11—C10—H10	119.3	C59—C58—C57	117.4 (4)
C9—C10—H10	119.3	C60—C59—C64	120.4 (5)
C10—C11—C12	121.1 (5)	C60—C59—C58	121.9 (4)
C10—C11—H11	119.4	C64—C59—C58	117.5 (4)
C12—C11—H11	119.4	C61—C60—C59	120.5 (5)
C11—C12—C13	119.0 (5)	C61—C60—H60	119.8
C11—C12—H12	120.5	C59—C60—H60	119.8
C13—C12—H12	120.5	C60—C61—C62	120.3 (5)
C12—C13—C14	121.1 (5)	C60—C61—H61	119.8
C12—C13—H13	119.5	C62—C61—H61	119.8
C14—C13—H13	119.5	C61—C62—C63	120.3 (5)
C13—C14—C9	120.0 (5)	C61—C62—H62	119.9
C13—C14—C1	121.5 (5)	C63—C62—H62	119.9
C9—C14—C1	118.3 (5)	C62—C63—C64	120.4 (5)
N3—C15—C28	127.5 (5)	C62—C63—H63	119.8
N3—C15—C16	112.3 (4)	C64—C63—H63	119.8
C28—C15—C16	120.1 (4)	C59—C64—C63	118.1 (5)
N4—C16—C17	127.5 (4)	C59—C64—C65	121.3 (4)
N4—C16—C15	112.2 (4)	C63—C64—C65	120.6 (4)
C17—C16—C15	120.2 (4)	C66—C65—C70	118.4 (5)
C18—C17—C22	119.4 (4)	C66—C65—C64	121.5 (4)
C18—C17—C16	122.8 (5)	C70—C65—C64	120.0 (4)
C22—C17—C16	117.7 (4)	C67—C66—C65	120.8 (5)
C19—C18—C17	121.0 (5)	C67—C66—H66	119.6
C19—C18—H18	119.5	C65—C66—H66	119.6
C17—C18—H18	119.5	C68—C67—C66	120.1 (5)
C20—C19—C18	119.4 (5)	C68—C67—H67	119.9
C20—C19—H19	120.3	C66—C67—H67	119.9
C18—C19—H19	120.3	C67—C68—C69	120.3 (5)
C21—C20—C19	120.4 (5)	C67—C68—H68	119.9
C21—C20—H20	119.8	C69—C68—H68	119.9
C19—C20—H20	119.8	C68—C69—C70	120.4 (5)
C20—C21—C22	121.8 (5)	C68—C69—H69	119.8
C20—C21—H21	119.1	C70—C69—H69	119.8
C22—C21—H21	119.1	C69—C70—C65	120.0 (5)
C21—C22—C17	117.9 (5)	C69—C70—C57	121.9 (4)
C21—C22—C23	120.6 (5)	C65—C70—C57	118.1 (4)
C17—C22—C23	121.3 (4)	C1—N1—O1	116.1 (4)
C24—C23—C28	117.1 (5)	C1—N1—Ni1	126.1 (3)
C24—C23—C22	121.3 (5)	O1—N1—Ni1	115.8 (3)
C28—C23—C22	121.6 (5)	C2—N2—O2	117.3 (4)
C25—C24—C23	122.7 (5)	C15—N3—O3	117.4 (4)
C25—C24—H24	118.6	C15—N3—Ni2	117.0 (3)
C23—C24—H24	118.6	O3—N3—Ni2	125.6 (3)
C24—C25—C26	119.7 (5)	O4—N4—C16	121.4 (4)
C24—C25—H25	120.2	O4—N4—Ni2	122.3 (3)
C26—C25—H25	120.2	C16—N4—Ni2	116.2 (3)
C25—C26—C27	119.6 (5)	C29—N5—O5	118.5 (4)
C25—C26—H26	120.2	C29—N5—Ni1	116.9 (4)
C27—C26—H26	120.2	O5—N5—Ni1	124.6 (3)
C26—C27—C28	121.1 (5)	C30—N6—O6	121.7 (4)
C26—C27—H27	119.5	C30—N6—Ni1	116.8 (3)
C28—C27—H27	119.5	O6—N6—Ni1	121.5 (3)
C27—C28—C23	119.7 (5)	C43—N7—O7	116.4 (4)
C27—C28—C15	123.0 (5)	C43—N7—Ni2	127.2 (4)
C23—C28—C15	117.3 (4)	O7—N7—Ni2	114.4 (3)
N5—C29—C30	112.0 (4)	C44—N8—O8	118.6 (4)
N5—C29—C42	127.8 (5)	C57—N9—O9	117.6 (4)
C30—C29—C42	120.3 (4)	C57—N9—Ni3	117.9 (3)
N6—C30—C31	126.9 (5)	O9—N9—Ni3	122.1 (3)
N6—C30—C29	112.3 (4)	C58—N10—O10	116.3 (4)
C31—C30—C29	120.9 (4)	C58—N10—Ni3	115.7 (3)
C32—C31—C36	118.8 (4)	O10—N10—Ni3	122.3 (3)
C32—C31—C30	123.2 (5)	N1—O1—B1	113.0 (4)
C36—C31—C30	118.0 (5)	N2—O2—Ni1	127.1 (3)
C33—C32—C31	121.8 (5)	N2—O2—Ni3	117.6 (3)
C33—C32—H32	119.1	Ni1—O2—Ni3	115.17 (16)
C31—C32—H32	119.1	N3—O3—B2	118.8 (4)
C32—C33—C34	119.7 (6)	N4—O4—Ni3	113.1 (3)
C32—C33—H33	120.1	N5—O5—B1	119.5 (4)
C34—C33—H33	120.1	N6—O6—Ni3	112.9 (3)
C35—C34—C33	119.5 (5)	N7—O7—B2	114.4 (4)
C35—C34—H34	120.2	N8—O8—Ni2	128.3 (3)
C33—C34—H34	120.2	N8—O8—Ni3	115.4 (3)
C34—C35—C36	123.6 (5)	Ni2—O8—Ni3	116.21 (17)
C34—C35—H35	118.2	N9—O9—H1A	109.5
C36—C35—H35	118.2	N10—O10—H2A	109.5
C35—C36—C31	116.6 (5)	O2—Ni1—N5	170.30 (16)
C35—C36—C37	122.7 (5)	O2—Ni1—N6	88.27 (16)
C31—C36—C37	120.7 (4)	N5—Ni1—N6	82.07 (18)
C38—C37—C42	118.0 (5)	O2—Ni1—N1	90.96 (16)
C38—C37—C36	119.4 (5)	N5—Ni1—N1	98.53 (18)
C42—C37—C36	122.6 (5)	N6—Ni1—N1	173.25 (18)
C39—C38—C37	121.8 (5)	O8—Ni2—N3	170.42 (16)
C39—C38—H38	119.1	O8—Ni2—N4	88.60 (16)
C37—C38—H38	119.1	N3—Ni2—N4	81.88 (17)
C38—C39—C40	120.3 (5)	O8—Ni2—N7	90.78 (17)
C38—C39—H39	119.8	N3—Ni2—N7	98.56 (19)
C40—C39—H39	119.8	N4—Ni2—N7	173.36 (18)
C39—C40—C41	119.4 (6)	O4—Ni3—O2	89.28 (14)
C39—C40—H40	120.3	O4—Ni3—N9	87.74 (15)
C41—C40—H40	120.3	O2—Ni3—N9	96.72 (16)
C42—C41—C40	120.9 (5)	O4—Ni3—O6	165.12 (14)
C42—C41—H41	119.6	O2—Ni3—O6	79.77 (13)
C40—C41—H41	119.6	N9—Ni3—O6	103.43 (16)
C41—C42—C37	119.6 (5)	O4—Ni3—O8	81.29 (14)
C41—C42—C29	123.0 (5)	O2—Ni3—O8	93.48 (15)
C37—C42—C29	117.4 (5)	N9—Ni3—O8	164.91 (17)
N7—C43—C56	127.1 (6)	O6—Ni3—O8	89.28 (14)
N7—C43—C44	117.3 (5)	O4—Ni3—N10	107.36 (16)
C56—C43—C44	115.5 (5)	O2—Ni3—N10	161.43 (17)
N8—C44—C43	126.5 (5)	N9—Ni3—N10	76.28 (17)
N8—C44—C45	114.4 (5)	O6—Ni3—N10	85.12 (16)
C43—C44—C45	119.1 (5)	O8—Ni3—N10	97.08 (16)
C50—C45—C46	120.8 (6)	F2—B1—F1	112.4 (5)
C50—C45—C44	118.7 (7)	F2—B1—O5	105.9 (5)
C46—C45—C44	120.5 (5)	F1—B1—O5	111.2 (5)
C47—C46—C45	121.0 (7)	F2—B1—O1	106.8 (5)
C47—C46—H46	119.5	F1—B1—O1	108.5 (5)
C45—C46—H46	119.5	O5—B1—O1	112.0 (5)
C46—C47—C48	118.5 (9)	F4—B2—F3	112.3 (5)
C46—C47—H47	120.7	F4—B2—O3	110.4 (5)
C48—C47—H47	120.7	F3—B2—O3	105.4 (5)
C49—C48—C47	120.5 (7)	F4—B2—O7	109.1 (5)
C49—C48—H48	119.7	F3—B2—O7	106.1 (5)
C47—C48—H48	119.7	O3—B2—O7	113.4 (5)
C48—C49—C50	121.7 (8)		
			
N1—C1—C2—N2	−33.6 (7)	C54—C55—C56—C43	176.8 (5)
C14—C1—C2—N2	146.8 (5)	C52—C51—C56—C55	−1.5 (8)
N1—C1—C2—C3	146.9 (5)	C50—C51—C56—C55	176.8 (5)
C14—C1—C2—C3	−32.8 (6)	C52—C51—C56—C43	−176.3 (5)
N2—C2—C3—C4	22.9 (7)	C50—C51—C56—C43	2.0 (8)
C1—C2—C3—C4	−157.5 (5)	N7—C43—C56—C55	24.5 (8)
N2—C2—C3—C8	−157.1 (5)	C44—C43—C56—C55	−157.4 (5)
C1—C2—C3—C8	22.6 (7)	N7—C43—C56—C51	−160.7 (5)
C8—C3—C4—C5	0.1 (8)	C44—C43—C56—C51	17.4 (7)
C2—C3—C4—C5	−179.8 (5)	N9—C57—C58—N10	−28.2 (6)
C3—C4—C5—C6	−0.2 (9)	C70—C57—C58—N10	152.2 (4)
C4—C5—C6—C7	−0.2 (10)	N9—C57—C58—C59	145.3 (4)
C5—C6—C7—C8	0.7 (10)	C70—C57—C58—C59	−34.4 (6)
C6—C7—C8—C3	−0.7 (8)	N10—C58—C59—C60	17.3 (8)
C6—C7—C8—C9	179.0 (5)	C57—C58—C59—C60	−154.5 (5)
C4—C3—C8—C7	0.3 (8)	N10—C58—C59—C64	−168.3 (5)
C2—C3—C8—C7	−179.7 (5)	C57—C58—C59—C64	19.9 (6)
C4—C3—C8—C9	−179.5 (5)	C64—C59—C60—C61	−1.7 (8)
C2—C3—C8—C9	0.5 (7)	C58—C59—C60—C61	172.6 (5)
C7—C8—C9—C14	166.8 (5)	C59—C60—C61—C62	1.4 (9)
C3—C8—C9—C14	−13.4 (7)	C60—C61—C62—C63	−0.8 (9)
C7—C8—C9—C10	−15.0 (8)	C61—C62—C63—C64	0.3 (8)
C3—C8—C9—C10	164.8 (5)	C60—C59—C64—C63	1.2 (7)
C14—C9—C10—C11	−0.2 (7)	C58—C59—C64—C63	−173.3 (4)
C8—C9—C10—C11	−178.5 (5)	C60—C59—C64—C65	179.8 (4)
C9—C10—C11—C12	1.3 (8)	C58—C59—C64—C65	5.3 (6)
C10—C11—C12—C13	−1.1 (8)	C62—C63—C64—C59	−0.6 (7)
C11—C12—C13—C14	−0.1 (8)	C62—C63—C64—C65	−179.2 (5)
C12—C13—C14—C9	1.2 (7)	C59—C64—C65—C66	164.0 (5)
C12—C13—C14—C1	175.8 (5)	C63—C64—C65—C66	−17.5 (7)
C10—C9—C14—C13	−1.0 (7)	C59—C64—C65—C70	−17.0 (7)
C8—C9—C14—C13	177.2 (5)	C63—C64—C65—C70	161.6 (5)
C10—C9—C14—C1	−175.8 (4)	C70—C65—C66—C67	0.5 (7)
C8—C9—C14—C1	2.5 (7)	C64—C65—C66—C67	179.6 (4)
N1—C1—C14—C13	26.0 (7)	C65—C66—C67—C68	−0.3 (8)
C2—C1—C14—C13	−154.4 (5)	C66—C67—C68—C69	0.0 (8)
N1—C1—C14—C9	−159.3 (5)	C67—C68—C69—C70	0.0 (8)
C2—C1—C14—C9	20.3 (6)	C68—C69—C70—C65	0.2 (8)
N3—C15—C16—N4	−5.2 (6)	C68—C69—C70—C57	178.7 (5)
C28—C15—C16—N4	173.2 (4)	C66—C65—C70—C69	−0.5 (7)
N3—C15—C16—C17	170.7 (4)	C64—C65—C70—C69	−179.5 (4)
C28—C15—C16—C17	−10.9 (7)	C66—C65—C70—C57	−179.1 (4)
N4—C16—C17—C18	−3.1 (8)	C64—C65—C70—C57	1.9 (7)
C15—C16—C17—C18	−178.4 (4)	N9—C57—C70—C69	24.8 (8)
N4—C16—C17—C22	174.2 (5)	C58—C57—C70—C69	−155.6 (5)
C15—C16—C17—C22	−1.0 (7)	N9—C57—C70—C65	−156.6 (5)
C22—C17—C18—C19	−1.5 (8)	C58—C57—C70—C65	23.0 (6)
C16—C17—C18—C19	175.9 (5)	C2—C1—N1—O1	−173.7 (4)
C17—C18—C19—C20	−1.3 (9)	C14—C1—N1—O1	6.0 (7)
C18—C19—C20—C21	2.5 (9)	C2—C1—N1—Ni1	23.2 (6)
C19—C20—C21—C22	−1.1 (9)	C14—C1—N1—Ni1	−157.2 (4)
C20—C21—C22—C17	−1.7 (8)	C3—C2—N2—O2	−175.1 (4)
C20—C21—C22—C23	174.2 (5)	C1—C2—N2—O2	5.3 (7)
C18—C17—C22—C21	2.9 (7)	C28—C15—N3—O3	2.9 (7)
C16—C17—C22—C21	−174.6 (5)	C16—C15—N3—O3	−178.9 (4)
C18—C17—C22—C23	−172.9 (4)	C28—C15—N3—Ni2	−176.9 (4)
C16—C17—C22—C23	9.6 (7)	C16—C15—N3—Ni2	1.4 (5)
C21—C22—C23—C24	−5.0 (8)	C17—C16—N4—O4	9.2 (7)
C17—C22—C23—C24	170.7 (5)	C15—C16—N4—O4	−175.3 (4)
C21—C22—C23—C28	177.8 (5)	C17—C16—N4—Ni2	−168.8 (4)
C17—C22—C23—C28	−6.6 (7)	C15—C16—N4—Ni2	6.8 (5)
C28—C23—C24—C25	2.0 (8)	C30—C29—N5—O5	−178.1 (4)
C22—C23—C24—C25	−175.3 (5)	C42—C29—N5—O5	0.8 (8)
C23—C24—C25—C26	0.8 (9)	C30—C29—N5—Ni1	1.0 (5)
C24—C25—C26—C27	−2.5 (9)	C42—C29—N5—Ni1	179.9 (4)
C25—C26—C27—C28	1.4 (8)	C31—C30—N6—O6	1.7 (7)
C26—C27—C28—C23	1.5 (8)	C29—C30—N6—O6	−178.3 (4)
C26—C27—C28—C15	−178.9 (5)	C31—C30—N6—Ni1	−178.0 (4)
C24—C23—C28—C27	−3.1 (7)	C29—C30—N6—Ni1	2.0 (5)
C22—C23—C28—C27	174.2 (5)	C56—C43—N7—O7	2.8 (8)
C24—C23—C28—C15	177.3 (5)	C44—C43—N7—O7	−175.3 (4)
C22—C23—C28—C15	−5.4 (7)	C56—C43—N7—Ni2	−160.1 (4)
N3—C15—C28—C27	12.4 (8)	C44—C43—N7—Ni2	21.8 (7)
C16—C15—C28—C27	−165.7 (5)	C43—C44—N8—O8	4.6 (8)
N3—C15—C28—C23	−168.0 (5)	C45—C44—N8—O8	−175.8 (4)
C16—C15—C28—C23	13.9 (7)	C70—C57—N9—O9	0.4 (8)
N5—C29—C30—N6	−1.9 (6)	C58—C57—N9—O9	−179.2 (4)
C42—C29—C30—N6	179.1 (4)	C70—C57—N9—Ni3	−162.4 (4)
N5—C29—C30—C31	178.1 (4)	C58—C57—N9—Ni3	18.0 (5)
C42—C29—C30—C31	−0.9 (7)	C59—C58—N10—O10	7.1 (8)
N6—C30—C31—C32	−0.6 (8)	C57—C58—N10—O10	179.3 (4)
C29—C30—C31—C32	179.4 (5)	C59—C58—N10—Ni3	−147.0 (4)
N6—C30—C31—C36	179.1 (5)	C57—C58—N10—Ni3	25.2 (5)
C29—C30—C31—C36	−0.9 (7)	C1—N1—O1—B1	137.4 (5)
C36—C31—C32—C33	2.2 (8)	Ni1—N1—O1—B1	−57.6 (5)
C30—C31—C32—C33	−178.1 (5)	C2—N2—O2—Ni1	32.8 (6)
C31—C32—C33—C34	−1.0 (9)	C2—N2—O2—Ni3	−142.9 (4)
C32—C33—C34—C35	−0.1 (10)	C15—N3—O3—B2	−175.3 (5)
C33—C34—C35—C36	0.0 (10)	Ni2—N3—O3—B2	4.4 (7)
C34—C35—C36—C31	1.2 (8)	C16—N4—O4—Ni3	−145.0 (4)
C34—C35—C36—C37	179.5 (6)	Ni2—N4—O4—Ni3	32.9 (4)
C32—C31—C36—C35	−2.2 (7)	C29—N5—O5—B1	−173.4 (5)
C30—C31—C36—C35	178.1 (5)	Ni1—N5—O5—B1	7.6 (7)
C32—C31—C36—C37	179.4 (4)	C30—N6—O6—Ni3	−146.0 (4)
C30—C31—C36—C37	−0.3 (7)	Ni1—N6—O6—Ni3	33.6 (4)
C35—C36—C37—C38	4.1 (7)	C43—N7—O7—B2	139.1 (5)
C31—C36—C37—C38	−177.6 (4)	Ni2—N7—O7—B2	−55.7 (5)
C35—C36—C37—C42	−174.7 (5)	C44—N8—O8—Ni2	29.2 (6)
C31—C36—C37—C42	3.6 (7)	C44—N8—O8—Ni3	−146.7 (4)
C42—C37—C38—C39	1.9 (8)	N2—O2—Ni1—N6	152.7 (4)
C36—C37—C38—C39	−177.0 (5)	Ni3—O2—Ni1—N6	−31.5 (2)
C37—C38—C39—C40	−0.1 (9)	N2—O2—Ni1—N1	−34.0 (4)
C38—C39—C40—C41	−0.6 (8)	Ni3—O2—Ni1—N1	141.8 (2)
C39—C40—C41—C42	−0.6 (8)	C29—N5—Ni1—N6	0.0 (4)
C40—C41—C42—C37	2.4 (8)	O5—N5—Ni1—N6	179.0 (4)
C40—C41—C42—C29	−176.4 (5)	C29—N5—Ni1—N1	−173.2 (4)
C38—C37—C42—C41	−3.0 (7)	O5—N5—Ni1—N1	5.8 (4)
C36—C37—C42—C41	175.8 (4)	C30—N6—Ni1—O2	177.9 (4)
C38—C37—C42—C29	175.9 (4)	O6—N6—Ni1—O2	−1.7 (3)
C36—C37—C42—C29	−5.2 (7)	C30—N6—Ni1—N5	−1.2 (4)
N5—C29—C42—C41	4.0 (8)	O6—N6—Ni1—N5	179.1 (4)
C30—C29—C42—C41	−177.2 (4)	C1—N1—Ni1—O2	4.2 (4)
N5—C29—C42—C37	−174.9 (5)	O1—N1—Ni1—O2	−159.1 (3)
C30—C29—C42—C37	3.9 (7)	C1—N1—Ni1—N5	−177.8 (4)
N7—C43—C44—N8	−29.8 (8)	O1—N1—Ni1—N5	19.0 (4)
C56—C43—C44—N8	151.8 (5)	N8—O8—Ni2—N4	157.2 (4)
N7—C43—C44—C45	150.5 (5)	Ni3—O8—Ni2—N4	−27.0 (2)
C56—C43—C44—C45	−27.8 (7)	N8—O8—Ni2—N7	−29.5 (4)
N8—C44—C45—C50	−161.0 (5)	Ni3—O8—Ni2—N7	146.4 (2)
C43—C44—C45—C50	18.7 (7)	C15—N3—Ni2—N4	1.7 (4)
N8—C44—C45—C46	18.7 (8)	O3—N3—Ni2—N4	−178.0 (4)
C43—C44—C45—C46	−161.6 (5)	C15—N3—Ni2—N7	−171.6 (4)
C50—C45—C46—C47	−0.2 (9)	O3—N3—Ni2—N7	8.7 (4)
C44—C45—C46—C47	−179.9 (6)	O4—N4—Ni2—O8	−3.9 (3)
C45—C46—C47—C48	−0.4 (11)	C16—N4—Ni2—O8	174.1 (4)
C46—C47—C48—C49	0.8 (12)	O4—N4—Ni2—N3	177.1 (4)
C47—C48—C49—C50	−0.6 (12)	C16—N4—Ni2—N3	−4.9 (3)
C46—C45—C50—C49	0.4 (9)	C43—N7—Ni2—O8	2.3 (5)
C44—C45—C50—C49	−179.9 (5)	O7—N7—Ni2—O8	−161.0 (3)
C46—C45—C50—C51	−178.3 (5)	C43—N7—Ni2—N3	−179.8 (5)
C44—C45—C50—C51	1.4 (8)	O7—N7—Ni2—N3	16.9 (4)
C48—C49—C50—C45	0.0 (10)	N5—O5—B1—F2	−161.0 (5)
C48—C49—C50—C51	178.7 (7)	N5—O5—B1—F1	76.6 (7)
C45—C50—C51—C52	166.2 (5)	N5—O5—B1—O1	−45.0 (7)
C49—C50—C51—C52	−12.4 (9)	N1—O1—B1—F2	−170.9 (4)
C45—C50—C51—C56	−12.1 (8)	N1—O1—B1—F1	−49.6 (6)
C49—C50—C51—C56	169.3 (6)	N1—O1—B1—O5	73.6 (6)
C56—C51—C52—C53	−0.8 (9)	N3—O3—B2—F4	80.1 (6)
C50—C51—C52—C53	−179.1 (6)	N3—O3—B2—F3	−158.4 (5)
C51—C52—C53—C54	2.5 (10)	N3—O3—B2—O7	−42.7 (7)
C52—C53—C54—C55	−1.9 (10)	N7—O7—B2—F4	−50.1 (6)
C53—C54—C55—C56	−0.4 (9)	N7—O7—B2—F3	−171.4 (4)
C54—C55—C56—C51	2.1 (8)	N7—O7—B2—O3	73.4 (6)

### (2) Bis(µ2-bis{[10-(oxidoimino)-9,10-dihydrophenanthren-9-ylidene]amino}difluoroborato)(phenanthrene-9,10-dione dioxime)trinickel(II) dichloromethane trisolvate Hydrogen-bond geometry (Å, º)

**Table:**

*D*—H···*A*	*D*—H	H···*A*	*D*···*A*	*D*—H···*A*
O9—H1*A*···N2	0.85	1.96	2.771 (6)	158
O10—H2*A*···N8	1.06	1.77	2.765 (6)	155
